# Unravelling co-mutational patterns with prognostic implications in *NPM1* mutated adult acute myeloid leukemia – a HARMONY study

**DOI:** 10.1038/s41375-025-02851-9

**Published:** 2026-01-14

**Authors:** Alberto Hernández-Sánchez, Ángela Villaverde Ramiro, Eric Sträng, Amin T. Turki, María Abáigar, Jurjen Versluis, Ian Thomas, Marta Sobas, Javier Martínez Elicegui, Gastone Castellani, Axel Benner, Raúl Azibeiro, Jesse M. Tettero, Rabea Mecklenbrauck, Joaquín Martínez-López, Marta Pratcorona, Ken I. Mills, Guillermo Sanz, Maria Teresa Voso, Lehmann Sören, Christoph Röllig, Christian Thiede, Klaus H. Metzeler, Konstanze Döhner, Michael Heuser, Torsten Haferlach, Peter JM Valk, Nigel Russell, Jesús María Hernández-Rivas, Brian Huntly, Gert Ossenkoppele, Hartmut Döhner, Lars Bullinger

**Affiliations:** 1 Hematology Department, University Hospital of Salamanca, Salamanca, Spain; 2 Institute of Biomedical Research of Salamanca (IBSAL), Salamanca, Spain; 3 Cancer Research Center of Salamanca (USAL-CSIC), Salamanca, Spain; 4 HARMONY Alliance Foundation, Salamanca, Spain; 5 Charité Universitätsmedizin Berlin, Berlin, Germany; 6 Marienhospital University Hospital, Ruhr-University Bochum, Bochum, Germany; 7 Institute for AI in Medicine, University Hospital Essen, Essen, Germany; 8 Erasmus MC Cancer Institute, University Medical Center Rotterdam, Rotterdam, the Netherlands; 9 Centre for Trials Research, Cardiff University, Cardiff, UK; 10 Department of Hematology, Collegium Medicum in Bydgoszcz, Nicolaus Copernicus University in Toruń, Bydgoszcz, Poland; 11 Department of Medical and Surgical Sciences (DIMEC), University of Bologna, Bologna, Italy; 12 IRCCS Azienda Ospedaliero-Universitaria di Bologna S.Orsola, Bologna, Italy; 13 Division of Biostatistics, German Cancer Research Center (DKFZ), Heidelberg, Germany; 14 Department of Hematology, Amsterdam UMC location VUMC, Amsterdam, Netherlands; 15 Department of Hematology, Hemostasis, Oncology and Stem Cell Transplantation, Hannover Medical School, Hannover, Germany; 16 Weatherall Institute of Molecular Medicine, University of Oxford, Oxford, UK; 17 Hospital Universitario 12 de Octubre, Madrid, Spain; 18 Hematology, Hospital de la Santa Creu i Sant Pau, Barcelona, Spain; 19 Patrick G Johnston Centre for Cancer Research, Queen’s University Belfast, Belfast, UK; 20 CIBERONC, Instituto de Salud Carlos III, Madrid, Spain; 21 Hospital Universitario y Politécnico La Fe, Valencia, Spain; 22 University of Rome Tor Vergata, Rome, Italy; 23 Uppsala University Hospital, Uppsala, Sweden; 24 Department of Medicine, Huddinge, Karolinska Institute, Stockholm, Sweden; 25 Medical Dept. I, University Hospital TU Dresden, Dresden, Germany; 26 University of Leipzig, Leipzig, Germany; 27 Department of Internal Medicine III, University Hospital of Ulm, Ulm, Germany; 28 Department of Internal Medicine IV, University Hospital Halle (Saale), Martin-Luther-University Halle-Wittenberg, Halle, Germany; 29 MLL Munich Leukemia Laboratory, Munich, Germany; 30 Guy’s and St Thomas’ Hospital, London, UK; 31 Wellcome-MRC Cambridge Stem Cell Institute, University of Cambridge, Cambridge, UK; 32 Department of Hematology, Oncology, and Tumor Immunology, Charité - Universitätsmedizin Berlin, Berlin, Germany; 33 German Cancer Consortium (DKTK), partner site Berlin, and German Cancer Research Center (DKFZ), Heidelberg, Germany

**Keywords:** Acute myeloid leukaemia, Acute myeloid leukaemia

## Abstract

*NPM1*-mutated (*NPM1*-mut) acute myeloid leukemia (AML) is generally associated with a more favorable outcome, although the presence of additional gene mutations can influence patient prognosis. We analyzed intensively-treated adult *NPM1*-mut AML patients included in the HARMONY Alliance database. A newly developed risk classification, which included combinations of co-mutations in *FLT3*-ITD, *DNMT3A*, *IDH1/IDH2*, and *TET2* genes, was applied to a training cohort of *NPM1*-mut AML patients included in clinical trials (*n* = 1001), an internal validation cohort more representative of real-world settings (*n* = 762), and an external validation cohort enrolled in UK-NCRI trials (*n* = 585). The HARMONY classification considered 51.8% of the *NPM1*-mut AML training cohort patients as favorable, 24.8% as intermediate, and 23.4% as adverse risk, with median overall survival (OS) of 14.4, 2.2, and 0.9 years, respectively; *p* < 0.001), thereby reclassifying 42.7% of *NPM1*-mut patients into a different European LeukemiaNet (ELN) 2022 risk category. These results were confirmed both in an internal and external validation cohort. Allogeneic hematopoietic stem cell transplantation (allo-HSCT) in first complete remission (CR1) showed the highest benefit in the *NPM1*-mut adverse-risk subgroup. The HARMONY classification provides the basis for a refined genetic risk stratification for adult *NPM1*-mut AML with potential clinical impact on allo-HSCT decision-making.

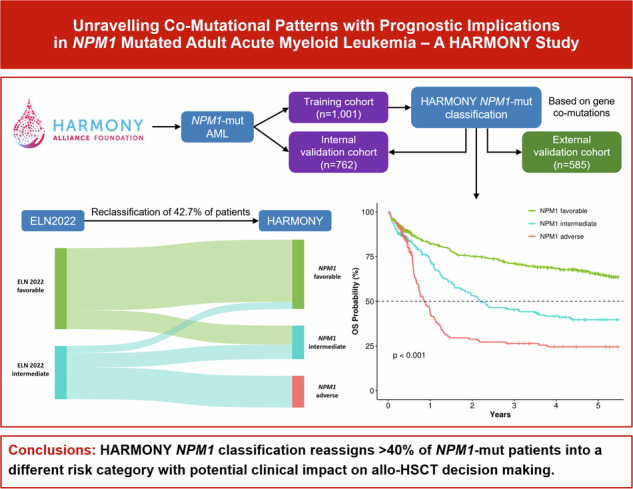

## Introduction

Acute myeloid leukemia (AML) is a clinically heterogeneous disease, where genomic alterations provide crucial prognostic insights that inform clinical decision-making [1]. *NPM1* mutations (*NPM1*-mut) have been described in approximately 30% of adult AML and define the largest disease subtype in younger adults, with distinct biologic and clinical features [2–4]. While the prognosis is generally considered favorable, a significant variability in outcomes has been reported. In fact, the vast majority of patients present several co-mutations that could influence the prognosis, such as *FLT3* internal tandem duplication (ITD), which has been recognized as a deleterious mutation. However, for almost two decades *FLT3*-ITD is the only co-mutation that is considered for risk stratification in *NPM1*-mut AML in current guidelines [5, 6]. Accordingly, in the European LeukemiaNet (ELN) 2022 risk classification, patients with *NPM1* mutations are categorized as favorable risk when *FLT3*-ITD is absent, but as intermediate risk when *FLT3*-ITD is also present [6]. Nevertheless, there are several other genes frequently co-mutated with *NPM1* that might influence the prognosis of this AML subtype. For example, *DNMT3A*-mut is present in around 50% of *NPM1*-mut AML, and it has been associated with adverse outcomes in several studies [7–11], although others found contradictory results [12]. In fact, the prognostic impact of *DNMT3A*-mut seems to be modulated by the frequent co-mutation of *FLT3*-ITD [13, 14]. This “triple-mutated” AML (*NPM1*-mut, *FLT3*-ITD, and *DNMT3A*-mut) is a significant group as it represents approximately 25% of *NPM1*-mut AML. On the other hand, the relatively poor outcomes attributed to *FLT3*-ITD might be influenced by the frequent co-mutation with *DNMT3A* [15]. Additional gene mutations have been reported in *NPM1*-mut AML, although their prognostic impact remains unclear [16].

Cytogenetic aberrations are uncommon in this AML subtype, where a normal karyotype has been reported in 80-88% of the patients [17–21]. Most of them do not seem to affect the risk stratification, with the exception of infrequent (<3%) adverse cytogenetic abnormalities according to ELN2022 consensus [6, 22]. Moreover, an aberrant karyotype does not seem to influence the immunophenotype nor gene expression profile in *NPM1*-mut AML, whereas they could be related to concomitant gene mutations [15, 19, 23, 24].

Hence, this AML subtype is an ideal setting for analyzing complex gene-gene interactions and co-mutational patterns with potential prognostic implications.

In order to address these uncertainties, we analyzed a large cohort of patients with *NPM1*-mut AML included in the Healthcare Alliance for Resourceful Medicine Offensive against Neoplasms in Hematology (HARMONY) AML international database and validated the findings using both HARMONY real-world data as well as publicly available data.

## Methods

### Patients

The HARMONY Alliance AML database was used for this study, where only patients fulfilling the following criteria were selected: age >18 years at AML diagnosis, presence of *NPM1*-mut, treatment with intensive chemotherapy regimens, availability of cytogenetic study, and next-generation sequencing (NGS) myeloid panel data. Patients who received targeted therapies (e.g., *FLT3* or *IDH1/IDH2* inhibitors, anti-CD33 antibodies) or non-intensive treatment approaches were not included. Patients with class-defining cytogenetic abnormalities concomitant with *NPM1*-mut were excluded.

A total of 1763 *NPM1*-mut AML adult patients were selected for this analysis, contributed by eight European centers or cooperative groups. The training cohort comprised 1001 *NPM1*-mut patients from three prospective multicenter clinical trials of the German–Austrian AML Study Group (AMLHD98A, AML-HD98B, and AMLSG-07-04) and from three prospective multicenter clinical trials of HOVON-SAKK (HO102, HO103, HO132), representing a clinical trial setting [1, 25–27]. An internal validation cohort was formed with the remainder of patients, contributed by the Study Alliance Leukemia AML registry (Germany), the Munich Leukemia Laboratory (Germany), the AML Cooperative Group registry (Germany), the Swedish AML registry (Sweden), the Hospital Universitario 12 de Octubre (Spain), and the Queen’s University of Belfast (North Ireland), with a total of 762 *NPM1*-mut patients that were more representative of the “real-world” setting in Europe [28].

Patient data uploaded to the HARMONY Big Data Platform underwent a rigorous double brokerage pseudonymization process, adhering to the General Data Protection Regulation (GDPR). Subsequently, the data were harmonized and converted using the Observational Medical Outcomes Partnership (OMOP) Common Data Model [29].

The study was performed in accordance with the Declaration of Helsinki and received approval from the HARMONY steering committee and AML working group. The HARMONY research project underwent review and approval by the Medicinal Research Ethics Committee of the University of Salamanca (PI 2018 10 128). HARMONY has established an ethical and data-protection framework for the secondary use of data, including *de facto* anonymization. Prior written informed consent had been obtained from all patients at respective HARMONY partner institutions.

### *NPM1*-mut risk stratification

A multi-step analysis of clinically significant gene co-mutations associated to *NPM1*-mut was performed. At each step, combinations of up to two additional genes (either mutated or wildtype) were explored. The 2-year overall survival (OS) for each combination was estimated using 100-fold bootstrap sampling and compared to the 2-year OS of *NPM1* wildtype (-wt) patients in the same dataset (German–Austrian AML Study Group and HOVON-SAKK clinical trials, n = 2473). Gene mutation combinations that allowed patient reclassification into different risk categories were selected. The classification included only genes that were analyzed in both NGS panels of the training cohort (Table S1), provided that each gene mutation was found in at least 10 patients (≥1% of the cohort). While *IDH2*-R172K mutation has proven to be associated with distinct outcomes when compared to other *IDH2*-mut and *IDH1*-mut, it is also mutually exclusive to *NPM1*-mut and therefore rarely found in this AML subtype (Table S2) [11, 30]. Moreover, exploratory analyses demonstrated similar findings with *IDH1*-mut and *IDH2*-mut in *NPM1*-mut (Figs. S1 and S2), so they were combined as *IDH*-mut (any mutated) or *IDH*-wt (both wildtype) in the final risk classification. The HARMONY *NPM1*-mut classification was tested in the aforementioned internal validation cohort.

### External validation dataset

An external validation was also performed, using the publicly available dataset published by Tazi et al., comprising AML adult patients enrolled in UK-NCRI trials (AML17, AML16, AML11, AML12, AML14, and AML15) who were not included in HARMONY at the time of the analysis [31]. Patients with *NPM1*-mut, treated intensively, with cytogenetic and NGS myeloid panel information, were selected for HARMONY classification validation. Of note, some of these patients received gemtuzumab ozogamicin (GO) or *FLT3* inhibitor lestaurtinib in addition to intensive chemotherapy regimens, as part of AML17 randomizations.

### Statistical analysis

Clinical endpoints were defined as recommended by international guidelines [6]. Composite complete remission (CRc) was defined as either complete remission (CR) or CR with incomplete hematologic recovery (CRi). OS and relapse-free survival (RFS) were estimated using the Kaplan-Meier method, and differences between survival distributions were evaluated using the log-rank test. Patients who underwent allo-HSCT in first complete remission (CR1) were censored at transplant date for OS and RFS analyses in both the training and internal validation cohorts. The Cox proportional hazards model was used for multivariable survival analysis, including clinically-significant variables as well as HARMONY *NPM1*-mut classification. Imputation was not performed for missing values. Co-occurrence and mutual exclusivity were tested for gene mutations present in at least 3% of patients, calculating q-value as previously reported [32]. The relative order in which mutations were acquired was inferred using the Bradley-Terry method, using pairwise comparisons of sex-corrected variant allele frequencies. All reported p-values were two-sided at the conventional 5% significance level. Analyses were performed using R statistical software (v3.6.3).

## Results

### Patient characteristics of AML training cohort

The training cohort of 1001 adult *NPM1*-mut AML patients included 54% females. The median age at diagnosis was 53 years, and 73% were younger than 60 years (Table 1). A normal karyotype was observed in 87% of patients, and 39% had *FLT3*-ITD mutation at diagnosis. CRc after induction treatment was achieved in 87% of patients, while 4% died before response assessment (early-death). Allo-HSCT was performed in 34% patients (24% in CR1). Median follow-up was 6 years, with a median OS of 8.3 years.

**Table 1:** Baseline characteristics of *NPM1-*mut adult AML patients included in the HARMONY database, comparing the training cohort to the internal validation cohort.

	Training cohort (*n* = 1001)	Internal validation cohort (*n* = 762)	*p*-value
**Female sex**	543 (54.2%)	419 (55%)	0.7938
**Median age in years (range)**	52.9 (18–81)	57 (18 - 86)	<0.0001
Age ≥ 60 years	269 (26.8%)	320 (42%)	<0.0001
**AML type**			
De novo AML	963 (96.2%)	573 (90.5%)	<0.0001
Secondary AML	38 (3.8%)	60 (9.5%)	
Prior HM	15 (1.5%)	48 (7.6%)	<0.0001
Therapy-related AML	23 (2.3%)	12 (1.6%)	0.2811
**Hemoglobin (g/dL)**	8.9 [Q1 = 7.6, Q3 = 10.3]	9.2 [Q1 = 8.2, Q3 = 10.5]	0.0037
**WBC (x10^9/L)**	23.8 [Q1 = 6.9, Q3 = 62.8]	36.8 [Q1 = 13.4, Q3 = 85.6]	<0.0001
WBC > 100 ×10^9/L	136 (13.6%)	144 (20.4%)	
**Platelets (x10^9/L)**	66.5 [Q1 = 38, Q3 = 116]	66.5[Q1 = 38.2, Q3 = 111]	0.8798
**Bone marrow % of blasts**	75 [Q1 = 45, Q3 = 89]	72.75 [Q1 = 52, Q3 = 87]	0.9417
**ELN 2022**			
Favorable	601 (60%)	405 (53.2%)	0.0185
Intermediate	391 (39.1%)	346 (45.4%)	
Adverse	9 (0.9%)	11 (1.4%)	
***FLT3*-ITD**	393 (39.3%)	349 (45.8%)	0.0068
**Treatment response**			
CRc	874 (87.3%)	606 (79.5%)	<0.0001
Refractory	89 (8.9%)	95 (12.5%)	
Not evaluable (early death)	38 (3.8%)	61 (8%)	
**Early death**			
30-day mortality	38 (3.8%)	61 (8%)	0.0002
60-day mortality	62 (6.2%)	88 (11.5%)	<0.0001
**Allogeneic HSCT**	341 (34.1%)	215 (29.3%)	0.0419
In CR1	242 (24.2%)	130 (17.1%)	0.0104
In other situations	99 (9.9%)	85 (11.1%)	
**Median survival in years (95% CI)**	8.25 (5.14–9.77)	2.84 (2.06–4.09)	<0.0001

*AML* acute myeloid leukemia, *HM* hematological malignancy, *WBC* white blood count, *ELN* European LeukemiaNet, *ITD* internal tandem duplication, *CRc* composite complete remission, *HSCT* hematopoietic stem cell transplantation, *CR1* first complete remission, *CI* confidence interval.

### Mutational landscape of *NPM1*-mut AML training cohort

**Fig. 1:**
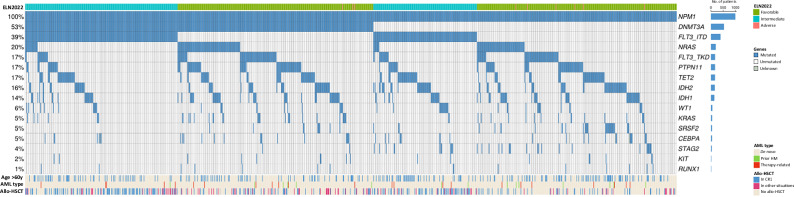
Mutational landscape of *NPM1*-mut adult AML. Only mutations present in both next-generation sequencing panels and in at least ten patients (1% of the training cohort) are shown. allo-HSCT allogeneic hematopoietic stem cell transplantation, CR1 first complete remission, ELN European LeukemiaNet, HM hematological malignancy.

In the training cohort, the most frequently mutated genes were *FLT3* in 54% (ITD 39%, TKD 17%), *DNMT3A* (53%), *NRAS* (20%), *PTPN11* (17%), *TET2* (17%), *IDH2* (16%), and *IDH1* (14%), while other gene mutations were present in less than 10% of patients (Fig. 1). Of note, 59.5% of patients with *FLT3*-ITD also had *DNMT3A*-mut, whereas 43.7% of *DNMT3A*-mut patients also presented with a *FLT3*-ITD, showing a strong co-occurrence of these two mutations in *NPM1*-mut AML (odds ratio [OR] 1.5, *q* = 0.014). Other significant co-occurring gene pairs, exploring all genes mutated in at least 3% of patients, were *IDH2* and *SRSF2* (OR 7.3, *q* < 0.001) as well as *IDH1* and *NRAS* (OR 1.8, *q* = 0.041). *FLT3*-ITD correlated negatively with *NRAS* (OR 0.2, *q* < 0.001), *KRAS* (OR 0.32, *q* = 0.008), *PTPN11* (OR 0.36, *q* < 0.001), and *SRSF2* (OR 0.1, *q* < 0.001). *DNMT3A* showed a negative correlation with *STAG2* (OR 0.05, *q* < 0.001) and *SRSF2* (OR 0.22, *q* < 0.001), while *IDH* correlated negatively with TET2 (*IDH1* OR 0.19, *q* < 0.001; *IDH2* OR 0.16, *q* < 0.001) and *WT1* (*IDH1* OR 0.2, *q* = 0.049; *IDH2* OR 0.18, *q* = 0.028). Based on the relative variant allele frequencies, mutations in *DNMT3A*, *STAG2*, *TET2*, *IDH1/2* and *SRSF2* appeared to represent early clonal events and generally arose prior to *NPM1*-mut, while mutations in genes associated with RAS signaling pathway were inferred to be acquired at later stages (Fig. S3).

### Development of the HARMONY risk classification for *NPM1*-mut AML

**Fig. 2:**
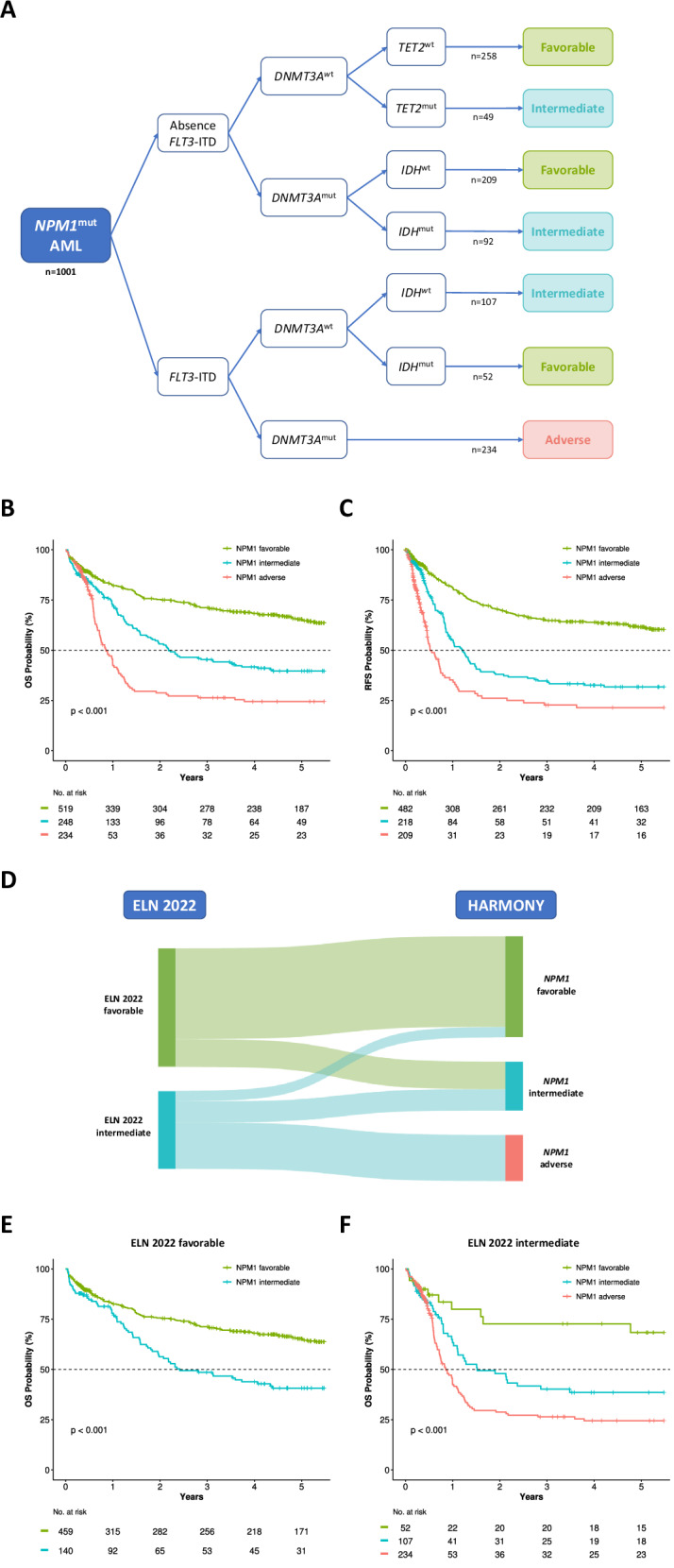
HARMONY *NPM1*-mut risk classification. **A** Classification of patients according to the presence or absence of mutations in *FLT3* (ITD), *DNMT3A*, *IDH* and *TET2*. **B** Overall survival according to HARMONY *NPM1*-mut risk categories. **C** Relapse-free survival according to HARMONY *NPM1*-mut risk categories. **D** Sankey plot of patient reclassification from ELN2022 to HARMONY *NPM1*-mut categories. **E** Overall survival of ELN2022 favorable patients, stratified by HARMONY *NPM1*-mut classification. **F** Overall survival of ELN2022 intermediate patients, stratified by HARMONY *NPM1*-mut classification.

In order to summarize clinically significant gene co-mutational patterns, a risk classification was developed using the *NPM1*-mut AML training cohort. Two-year OS of *NPM1*-wt intensively-treated patients in the same dataset was used as a reference and was 29.9% (95% CI 26.5–33.3%), 47.8% (42.4–53.3%), and 79% (74.3–83.7%) for European LeukemiaNet (ELN) 2022 adverse, intermediate, and favorable risk, respectively (Table S3). In the first step (all 1,001 *NPM1*-mut patients), co-mutation of *FLT3*-ITD and *DNMT3A*-mut was selected, as patients with both mutations had an estimated 2-year OS of 29.1% (21.6-36.6%), similar to ELN2022 adverse (Figs. S4–S7). Among patients with *FLT3*-ITD and *DNMT3A*-wt, those with *IDH*-mut had a predicted 2-year OS of 72.7% (56.4–89%), similar to the ELN2022 favorable-risk group, while *IDH*-wt patients showed an estimated 2-year OS of 47.4% (36.1–58.8%), comparable to the ELN2022 intermediate-risk group (Figs. S8 and S9). In accordance, 60% of patients with *FLT3*-ITD in *NPM1*-mut AML were classified as adverse, 27% as intermediate, and 13% as favorable risk (median OS 0.9 years, 1.5 years, and not reached, respectively, *p* < 0.001) (Fig. S10). In the next step, patients with the absence of *FLT3*-ITD were analyzed, and the combination of *DNMT3A* and *IDH* mutations was selected. Patients with *DNMT3A*-mut and *IDH*-mut had an estimated 2-year OS of 55.1% (43.6-66.5%), comparable to ELN2022 intermediate, while patients with *DNMT3A*-mut and *IDH*-wt presented a predicted 2-year OS of 77.2% (70.6-83.8%), similar to ELN2022 favorable (Figs. S11–S14). As a result, 44% of patients with *DNMT3A*-mut in *NPM1*-mut AML were classified as adverse, 17% as intermediate, and 39% as favorable risk (median OS 0.9, 2.3, and 9.5 years, respectively, *p* < 0.001) (Fig. S15). In the last step (absence of *FLT3*-ITD and *DNMT3A*-wt), patients with *TET2*-mut presented an estimated 2-year OS of 56.9% (41-72.8%), in line with ELN2022 intermediate, while patients with *TET2*-wt had a predicted 2-year OS of 73.5% (67.7-79.3%), closer to ELN2022 favorable (Figs. S16 and S17). Of note, the OS curve of *TET2*-mut patients did not present a plateau at the 2-year mark, which resulted in significant differences in OS when compared to *TET2*-wt patients (*p* = 0.022, Fig. S18). In the subgroup of patients with *FLT3*-ITD absence, *DNMT3*-wt and *TET2*-wt, further risk reclassification could not be made according to the classification requisites, resulting in those patients being categorized as *NPM1*-mut favorable (Figs. 2A, S19).

The HARMONY *NPM1*-mut risk classification stratified 51.8% of *NPM1*-mut AML patients as favorable, 24.8% as intermediate and 23.4% as adverse risk (median OS 14.4, 2.2 and 0.9 years, respectively, *p* < 0.001) (Fig. 2B). CRc rates after induction treatment were 90.7%, 83.5% and 83.7% for favorable, intermediate and adverse risk, respectively (*p* < 0.001). Median RFS was not reached for favorable risk, while it was 1.2 years for intermediate (95% CI 0.92–1.49) and 0.6 years for adverse (95% CI 0.46–0.74) (Fig. 2C). Of note, gender distribution, median patient age, AML type, hemoglobin and platelet values were similar among these three *NPM1*-mut risk categories (Table 2). *FLT3*-ITD was present in 10%, 43% and 100% of favorable, intermediate, and adverse risk patients, respectively, which could explain differences in WBC at diagnosis, bone marrow blasts and allo-HSCT rates in CR1 among the subgroups.

**Table 2:** Comparison of patient characteristics across HARMONY *NPM1*-mut risk categories.

	*NPM1* Favorable (*n* = 519)	*NPM1* Intermediate (*n* = 248)	*NPM1* Adverse (*n* = 234)	*p*-value
**Female sex**	275 (53%)	134 (54%)	134 (57.3%)	0.5500
**Median age in years (range)**	52.2 [18–81]	53.1 [18.8–80]	52.3 [24–77]	0.5290
Age ≥60 years	136 (26.2%)	72 (29%)	61 (26.1%)	0.6758
**AML type**				
De novo AML	497 (95.8%)	238 (96%)	228 (97.4%)	0.5249
Secondary AML	22 (4.2%)	10 (4%)	6 (2.6%)	
Prior HM	10 (1.9%)	4 (1.6%)	1 (0.4%)	0.2885
Therapy-related AML	12 (2.3%)	6 (2.4%)	5 (2.6%)	0.9783
**Hemoglobin (g/dL)**	9.15 [Q1 = 7.8, Q3 = 10.4]	8.89 [Q1 = 7.5, Q3 = 10]	8.65 [Q1 = 7.5, Q3 = 10.4]	0.2938
**WBC (x10^9/L)**	14.2 [Q1 = 4.7, Q3 = 42.5]	26.8 [Q1 = 8.6, Q3 = 71.2]	50.6 [Q1 = 22, Q3 = 93.9]	< 0.0001
WBC > 100 ×10^9/L	37 (7.1%)	44 (17.7%)	55 (23.5%)	< 0.0001
**Platelets (x10^9/L)**	68 [Q1 = 38, Q3 = 118]	67 [Q1 = 39, Q3 = 120.5]	63 [Q1 = 38, Q3 = 104]	0.5066
**Bone marrow % of blasts**	66 [Q1 = 36, Q3 = 86]	79.5 [Q1 = 52, Q3 = 90]	80 [Q1 = 63, Q3 = 90]	< 0.0001
**ELN 2022**				
Favorable	459 (88.4%)	141 (56.9%)	0 (0%)	< 0.0001
Intermediate	52 (10%)	106 (42.7%)	234 (100%)	
Adverse	8 (1.5%)	1 (0.4%)	0 (0%)	
***FLT3*-ITD present**	52 (10%)	107 (43.1%)	234 (100%)	< 0.0001
**Treatment response**				
CRc	471 (90.7%)	207 (83.5%)	196 (83.7%)	< 0.0001
Refractory	30 (5.8%)	30 (12.1%)	29 (12.5%)	
Not evaluable	18 (3.5%)	11 (4.4%)	9 (3.8%)	
**Early death**				
30-day mortality	18 (3.5%)	11 (4.4%)	9 (3.8%)	0.7317
60-day mortality	28 (5.4%)	21 (8.5%)	13 (5.6%)	0.2298
**Allogeneic HSCT**	131 (25.2%)	94 (37.9%)	116 (49.6%)	< 0.0001
In CR1	93 (17.9%)	56 (22.6%)	93 (39.8%)	0.0011
In other situations	38 (7.3%)	38 (15.3%)	23 (9.8%)	
**Median survival in years (95% CI)**	14.37 (9.51–NA)	2.56 (1.98–4.38)	1.13 (0.966–1.5)	< 0.001

*AML* acute myeloid leukemia, *HM* hematological malignancy, *WBC* white blood count, *ELN* European LeukemiaNet, *ITD* internal tandem duplication, *CRc* composite complete remission, *HSCT* hematopoietic stem cell transplantation, *CR1* first complete remission, *CI* confidence interval.

### Comparison to ELN 2022 risk classification

OS of *NPM1*-mut AML patients according to HARMONY categories was similar to reference ELN2022 subgroups in *NPM1*-wt in the training cohort: median OS 11.2 vs 14.4 years for favorable (*p* = 0.396), 1.7 vs 2.2 for intermediate (*p* = 0.386) and 1.1 vs 0.9 for adverse risk categories (*p* = 0.117) (Fig. S20). The HARMONY classification was able to reassign 42.7% of *NPM1*-mut patients into a different risk category: 234 shifted from intermediate to adverse, 141 from favorable to intermediate and 52 from intermediate to favorable (Fig. 2D, Table S4). Within the ELN2022 favorable subgroup, HARMONY *NPM1*-mut favorable patients had significant better outcomes than *NPM1*-mut intermediate cohort (median OS 14.4 vs 2.4 years, respectively, *p* < 0.001) (Fig. 2E). Within the ELN2022 intermediate group, the HARMONY classification was able to discriminate three different subgroups with distinct outcomes: *NPM1*-mut favorable, intermediate an adverse (median OS not reached, 1.5 and 0.9 years, respectively, *p* < 0.001) (Fig. 2F). The predictive performance of 5-year OS of HARMONY classification, measured by the time-dependent receiver operating curve (AUC(t)) was higher than that of ELN2022 (0.695 vs 0.635 respectively, Table S5).

The HARMONY classification was also able to stratify older patients (i.e.,>60 years at diagnosis) into three subgroups with distinct outcomes, with a median OS of 3.5, 1.1, and 0.6 years for favorable, intermediate, and adverse subgroups, respectively (*p* < 0.001) (Fig. S21).

### Multivariable analysis

**Fig. 3:**
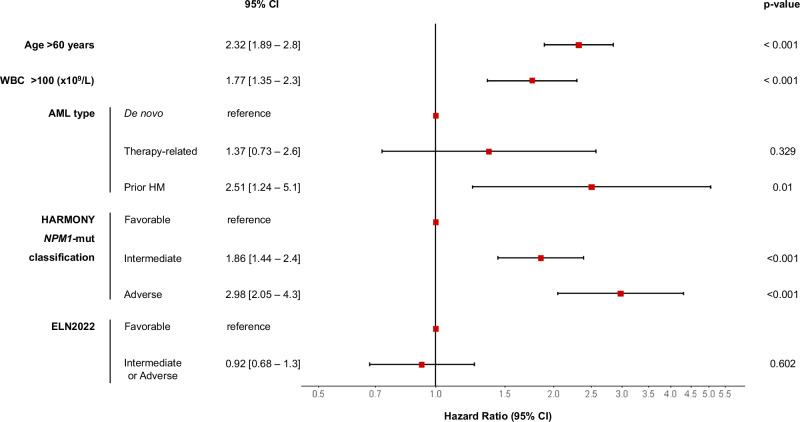
Multivariable Cox regression model of factors associated with OS. Patients that underwent allo-HSCT in CR1 were censored at the transplant date. HR hazard ratio, CI confidence interval, WBC white blood count, AML acute myeloid leukemia, HR hematological malignancy, mut mutated.

A multivariable Cox regression model of OS, censoring at transplant date those patients who underwent allo-HSCT in CR1, identified the following pretreatment independent variables: age >60 years (hazard ratio [HR] 2.32, *p* < 0.001), hyperleukocytosis (>100 ×10^9^/L) at diagnosis (HR 1.77, *p* < 0.001), prior hematological malignancy (HR 2.51, *p* = 0.01) and HARMONY *NPM1*-mut classification (using favorable category as reference, intermediate HR 1.86 [*p* < 0.001] and adverse HR 2.98 [*p* < 0.001]). Remarkably, ELN2022 risk categories were not significant in this model (*p* = 0.602) (Fig. 3).

Finally, a multivariable Cox regression model of OS, considering allo-HSCT in CR1 as a time-dependent covariate, in patients aged ≤70 years (i.e., potential transplant candidates) was performed. In the training cohort, allo-HSCT in CR1 improved OS in all HARMONY *NPM1*-mut subgroups, although the highest benefit was seen in *NPM1*-mut adverse patients (HR 0.66, 95% CI 0.57–0.77, *p* < 0.001) (Table S6).

### Other less frequent genomic abnormalities

The prognostic significance of additional gene mutations — those not included in the HARMONY *NPM1*-mut risk classification due to their low prevalence in the training cohort — was also investigated. *TP53*-mut (*n* = 7) was associated to poor outcomes, with a median OS of 1.2 years (compared to 6.2 for *TP53*-wt patients, *p* = 0.002) (Fig. S22). Similarly, *RUNX1*-mut was associated with shorter OS, especially in patients lacking *FLT3*-ITD (Fig. S23), while *SRSF2*-mut patients resembled intermediate prognosis, with a median OS of 2.4 years in that subset (Fig. S24). In contrast, *STAG2* or *RAD21* mutations were linked to improved OS (median OS not reached, Fig. S25). Notably, the presence of adverse-risk cytogenetic abnormalities (*n* = 9) did not correlate with distinct OS (Fig. S26).

### Internal validation of HARMONY *NPM1*-mut classification

The internal validation cohort of 762 adult *NPM1*-mut AML patients had significant differences compared to the training cohort, as patients were older (median 57 vs 53 years, *p* < 0.001; age ≥60 years 42% vs 27%, *p* < 0.001), with an increased proportion of patients with history of prior hematological malignancies (7.6% vs 1.5%), higher *FLT3*-ITD prevalence (45.8% vs 39.3%, *p* = 0.007) and increased WBC at diagnosis (37 vs 24 × 10^9^/L, *p* < 0.001) (Table 1). CRc rates after induction treatment were lower (79.5% vs 87.3%, *p* < 0.001), early-death rates were higher (30-day mortality 8% vs 3.8%, *p* < 0.001), and fewer patients received allo-HSCT in CR1 (17% vs 24%, *p* = 0.01). Median follow-up was 7.2 years, with a median OS of 2.8 years (vs 8.3 years in the training cohort, *p* < 0.001).

**Fig. 4:**
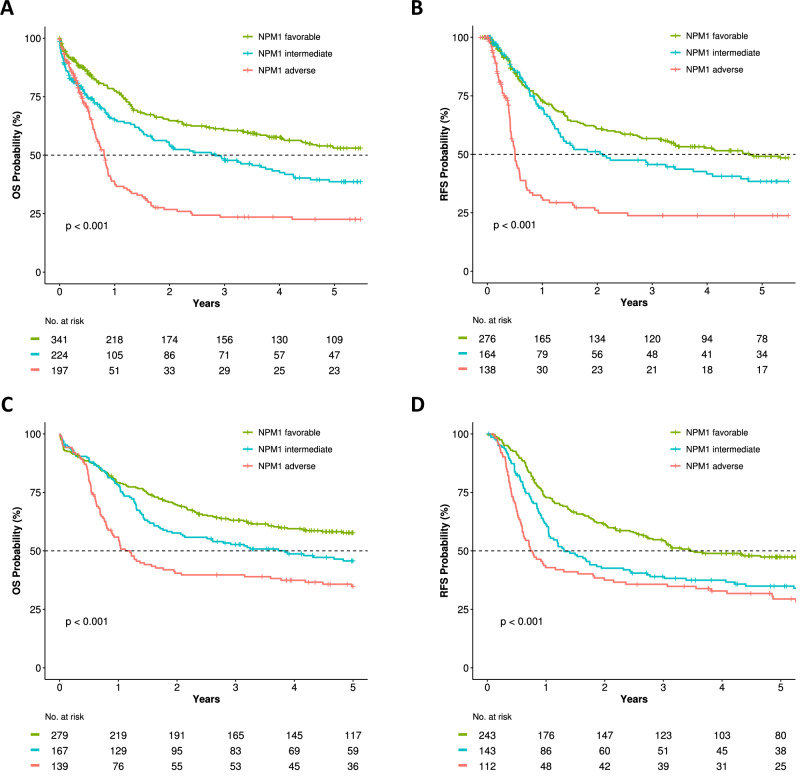
Stratification of outcomes in the validation cohorts according to HARMONY *NPM1*-mut risk categories. **A** OS in internal validation cohort, **B** RFS in internal validation cohort, **C** OS in external validation cohort, **D** RFS in external validation cohort.

The HARMONY *NPM1*-mut classification stratified 44.8% of the internal validation cohort as favorable, 29.4% as intermediate and 25.8% as adverse risk, with significant differences in OS (median OS 8.2, 2.8 and 0.8 years, respectively, *p* < 0.001) (Fig. 4A) and RFS (median RFS 4.8, 2.1 and 0.5 years, respectively, *p* < 0.001) (Fig. 4B). In patients aged >60 years at diagnosis, median OS was 3, 1.6 and 0.6 years, respectively (*p* < 0.001) (Fig. S21).

In the internal validation cohort, allo-HSCT in CR1 did not enhance OS of HARMONY *NPM1*-mut favorable patients (HR 0.99, 95% CI 0.94–1, *p* = 0.605), but it showed improved outcomes for the *NPM1*-mut intermediate subgroup (HR 0.84, 95% CI 0.75–0.94, *p* = 0.003) and again the highest benefit for *NPM1*-mut adverse patients (HR 0.78, 95% CI 0.69–0.88, *p* < 0.001) in the multivariable Cox regression model of OS, considering allo-HSCT in CR1 as a time-dependent covariate, in patients aged ≤70 years (Table S7).

### External validation of HARMONY *NPM1*-mut classification

The external validation cohort of 585 adult *NPM1*-mut AML patients also presented significant differences compared to the training cohort (Table S8). In the external validation cohort, patients were also older (median 56 vs 53 years, *p* < 0.001; age ≥60 years 38% vs 27%, *p* < 0.001), with an increased proportion of patients with history of prior hematological malignancies (4.8% vs 1.5%, *p* < 0.001), higher WBC at diagnosis (33 vs 24 ×10^9^/L, *p* < 0.001), but similar *FLT3*-ITD prevalence (41.5% vs 39.3%, *p* = 0.401). Composite complete remission rates after induction treatment were similar (89.6% vs 87.3%), although early death rate was higher (30-day mortality 6% vs 3.8%, *p* = 0.045). Allo-HSCT rates were higher (41.7% vs 34.1% at any time, *p* = 0.003), although information regarding the transplant timing was not provided for most of the patients. Therefore, OS and RFS were analyzed without censoring at transplant date in the external validation cohort. Median follow-up was 6.4 years, with a median OS of 4.5 years (vs 8.25 years in the training cohort, *p* < 0.001). In summary, the external validation cohort exhibited features that, in general, fell between those of the training cohort and the internal validation cohort (Table S9).

The HARMONY *NPM1*-mut classification stratified 47.7% of the external validation cohort as favorable, 28.5% as intermediate and 23.8% as adverse risk, with significant differences in OS (median OS not reached, 3.8 and 1.2 years, respectively, *p* < 0.001) (Fig. 4C) and RFS (median RFS 3.5, 1.3 and 0.8 years, respectively, *p* < 0.001) (Fig. 4D). AUC(t) of 5-year OS was 0.6 for HARMONY *NPM1*-mut classification and 0.558 for ELN2022 (Table S5). In patients aged >60 years at diagnosis, median OS was 3.6, 1.7, and 0.7 years, respectively (*p* < 0.001) (Supplementary Fig. S21).

## Discussion

*NPM1*-mut AML comprises the largest adult AML subtype in younger adults, making accurate risk stratification of paramount importance to inform clinical decisions [11]. Since the initial discovery of this entity, *FLT3*-ITD has been the only co-mutation consistently associated with inferior OS and remains the only significant co-mutation affecting prognosis in this AML subtype in current guidelines [2, 5, 6, 33–35]. In this study, which, to the best of our knowledge, is the largest *NPM1*-mut AML cohort studied by panel sequencing to date, we conducted a comprehensive analysis of the mutational landscape, identifying additional co-mutations with prognostic implications. *DNMT3A* and *FLT3*-ITD are the most frequent co-mutations, but they tend to appear together, making it difficult to address the prognostic value of each mutation individually in smaller cohorts. In our study, *FLT3*-ITD with *DNMT3A*-wt was present in only 16% of patients, *DNMT3A*-mut in the absence of *FLT3*-ITD was found in 30%, while both mutations were present in 23% of the patients, which is in line with recent reports [7]. This “triple-mutated” AML subgroup (*NPM1*-mut, *FLT3*-ITD, *DNMT3A*-mut) was associated with dismal outcomes, with a median RFS of less than 9 months in all datasets included in our study.

The interaction between these three mutations has been reported previously, consistently associated with inferior OS [1, 7, 13, 14, 36]. Moreover, recent studies have found that this triple-mutated AML shows distinct characteristics, such as aberrant leukemia-specific GPR56^high^ and CD34^low^ immunophenotype, high leukemia stem cell frequency, and upregulation of hepatic leukemia factor [15]. While it remains unclear if the *NPM1*-mut, *FLT3*-ITD, *DNMT3A*-mut subgroup will be recognized as a distinct biologic entity in the future, it seems reasonable to consider this subgroup as an adverse risk, at least with conventional chemotherapy approaches.

In addition to this important confirmatory aspect of our study, we also identified a subset of *FLT3*-ITD positive *NPM1*-mut patients with favorable outcomes (i.e. *NPM1*-mut, *FLT3*-ITD, *DNMT3A*-wt, and *IDH*-mut) that has not been reported in previous studies. Remarkably, *IDH*-mut were associated with a favorable outcome in patients with *FLT3*-ITD and *DNMT3A*-wt, while a deleterious effect was shown in the subgroup of patients with *DNMT3A*-mut and absence of *FLT3*-ITD, which is consistent with the results reported by Paschka et al. in the latter subgroup [37]. Moreover, this paradoxical prognostic effect has been documented for other gene mutations in adult AML, such as *DNMT3A* and *PTPN11*, highlighting the importance of careful evaluation of co-mutational patterns for accurate patient risk stratification [9, 38]. While the biological mechanisms underlying the impact of *IDH*-mut on treatment outcome in *NPM1*-mut AML remain to be fully elucidated, it could be related to the epigenetic state in which the transforming events occur, such as *NPM1*-mut and *FLT3*-ITD, as these events are generally acquired at a later stage [7, 39]. In contrast, the acquisition of *DNMT3A*-mut and *IDH*-mut is are early event, both linked to clonal hematopoiesis [40], and deregulated epigenetic states that are distinct between *DNMT3A*-mut and *IDH*-mut [41].

The incorporation of myelodysplasia (MDS)-related gene mutations (i.e., *ASXL1*, *BCOR*, *EZH2*, *RUNX1*, *SF3B1*, *SRSF2*, *STAG2*, *U2AF1*, *ZRSR2*) in the ELN2022 guidelines as adverse risk has raised the question of whether their presence could influence outcome in *NPM1*-mut AML, with contradictory results to date [42, 43]. We found that only three of these mutations (*SRSF2*, *STAG2*, *RUNX1*) were present in more than 1% of our cohort, which precluded the consideration of most MDS-related gene mutations into the classification. Notably, in our cohort, the presence of a *RUNX1*-mut was associated with adverse outcome, *SRSF2*-mut with intermediate prognosis, while *STAG2*-mut identified a subgroup of patients with favorable outcomes in the absence of *FLT3*-ITD. These findings suggest that MDS-related gene mutations, which are also early genetic events like clonal hematopoiesis-associated gene mutations, provide a different basis for transforming events, which, in the case of *NPM1*-mut can impact patient outcome in distinct ways. Moreover, the presence of adverse-risk cytogenetic abnormalities was not associated with inferior outcomes in our training cohort, while *TP53*-mut patients showed a significantly shorter OS. Both events are uncommon in *NPM1*-mut AML, so further studies will be required to confirm these clinically relevant findings.

In *NPM1*-mut AML, a median of up to 13 gene mutations per patient when whole exome analysis is performed has been reported, so hundreds of co-mutational combinations can form in this AML subtype [44]. In our study, we aimed to reduce this complexity in the final classification by taking only five of the most prevalent gene mutations into account: *FLT3*-ITD, *DNMT3A*-mut, *IDH1/IDH2*-mut, and *TET2*-mut. The HARMONY classification was able to stratify *NPM1*-mut patients into favorable, intermediate, and adverse risk categories, with comparable outcomes to their *NPM1*-wt counterparts according to ELN2022 in the same dataset. Remarkably, this risk stratification was able to reclassify more than 40% of *NPM1*-mut patients into a different risk category when compared to ELN2022 guidelines, underscoring the importance of understanding respective co-mutational patterns that can affect AML subtype-specific outcomes [6].

Importantly, the distribution of *NPM1*-mut risk categories was similar in all cohorts analyzed, both the training as well as the internal and external validation cohorts, accounting for 45-52% favorable, 25-29% intermediate, and 23-26% adverse risk patients contained within the *NPM1*-mut AML subtype. Prediction of outcomes in intensively-treated older patients (i.e.,>60 years) is challenging with current stratification systems, so novel approaches are warranted [45, 46]. The HARMONY classification was also applicable to older patients and, similarly to the entire cohort, identified three different outcome subgroups in the training as well as internal validation and external validation cohorts.

However, there are also several limitations to our study that should be taken into account. First, it is a retrospective, multicenter analysis, where patients were treated in various European countries with different intensive chemotherapy regimens. Accordingly, the HARMONY *NPM1*-mut classification was first developed in a cohort of patients included in clinical trials from large cooperative groups (AMLSG and HOVON-SAKK), thereby harboring a potential selection bias. However, findings could be validated in an independent validation cohort comprising real-world data derived from European institutions. Although this cohort did not include patients treated with intensive combination therapies including targeted therapeutic such as *FLT3*–ITD inhibitors or GO, which have demonstrated to improve outcomes in *NPM1*-mut AML [47–50], an additional validation of the HARMONY classification was carried out with a cohort of patients enrolled in UK-NCRI trials, where patients received GO or *FLT3* inhibitor lestaurtinib in addition to intensive chemotherapy. In accordance, further studies will be required to re-evaluate the prognostic and predictive impact of the HARMONY *NPM1*-mut classification to stratify patients treated with midostaurin or non-intensive approaches. Our analyses did not include assessment of measurable residual disease (MRD). While MRD in peripheral blood after two treatment cycles has proven to be a strong prognostic factor in *NPM1*-mut AML, novel treatment strategies have reduced the MRD positivity rate to less than 20% of patients, limiting the proportion of patients identified as high-risk [51, 52]. Moreover, a 3-year cumulative incidence of relapse of up to 40% has been reported even in MRD-negative patients [7], suggesting that a combination of genotype at diagnosis and MRD assessment after treatment would provide the most accurate estimation of patient outcomes.

In summary, this study provides novel insights regarding co-mutational patterns with prognostic implications in intensively-treated *NPM1*-mut AML adult patients. The HARMONY classification suggests that more than 40% of *NPM1*-mut patients might be re-classified into a different ELN2022 risk category, taking additional markers into account. Further evaluation is warranted prior to clinical application, especially in the light of age-dependent differences in co-mutational patterns [53] and novel combinatorial treatments for this AML subtype.

## Supplementary information

Supplementary appendix

## Data Availability

After the publication of this article, data collected for this analysis and related documents will be made available to others upon reasonably justified request, which needs to be written and addressed to the attention of the corresponding author, Dr Lars Bullinger at the following e-mail address: lars.bullinger@charite.de. The HARMONY Alliance, via the corresponding author Dr Lars Bullinger, is responsible to evaluate and eventually accept or refuse every request to disclose data and their related documents, in compliance with the ethical approval conditions, in compliance with applicable laws and regulations, and in conformance with the agreements in place with the involved subjects, the participating institutions, and all the other parties directly or indirectly involved in the participation, conduct, development, management, and evaluation of this analysis.
