# Supplementary material for: Unravelling co-mutational patterns with prognostic implications in NPM1 mutated adult acute myeloid leukemia – a HARMONY study
Source: Leukemia. 2026 Jan 14;40(2):418–28. doi: 10.1038/s41375-025-02851-9 (PMC12875867; doi:10.1038/s41375-025-02851-9)
Supplement: Supplementary file 1 — Supplementary appendix [file 41375_2025_2851_MOESM1_ESM.pdf]

Supplementary Appendix

to

Unravelling Co-Mutational Patterns with Prognostic Implications in *NPM1* Mutated Adult Acute Myeloid Leukemia – A HARMONY Study

by Hernández-Sánchez et al.

Table of contents

Supplementary methods 3

References 5

Supplementary tables 6

Table S1. List of genes included in both NGS panels of the training cohort. 6

Table S2. *IDH1* and *IDH2* mutation variants in the training cohort. 7

Table S3. Baseline characteristics training cohort, comparing patients with *NPM1* mutation (*NPM1*-mut) to patients with *NPM1* wildtype (*NPM1*-wt). 8

Table S4. Comparison of HARMONY *NPM1* and ELN2022 risk classification criteria for *NPM1*-mut AML. 9

Table S5. Predictive performance of 5-year OS of HARMONY *NPM1* risk classification compared to ELN2022, measured by the time-dependent receiver operating curve (AUC(t)). 10

Table S6. Univariable and multivariable analysis of overall survival in the training cohort, including allogeneic stem cell transplantation in first complete remission (allo-HSCT in CR1) as a time-dependent covariate. 11

Table S7. Univariable and multivariable analysis of overall survival in the internal validation cohort, including allogeneic stem cell transplantation in first complete remission (allo-HSCT in CR1) as a time-dependent covariate 12

Table S8. Comparison of baseline patient characteristics between training cohort and external validation cohort. 13

Table S9. Comparison of baseline patient characteristics between internal validation cohort and external validation cohort. 14

Supplementary figures 15

Figure S1. *IDH1/2* sub-analysis of overall survival in patients with *NPM1*-mut, absence *FLT3*-ITD and *DNMT3A*-mut. 15

Figure S3. Variant allele frequency of most-frequently mutated genes in *NPM1*-mut AML (training cohort) and Bradley-Terry model of relative order of mutation acquisition. 17

Figure S4. Two-year overall survival comparison of gene co-mutations in *NPM1*-mut AML compared to ELN2022 risk classification in *NPM1*-wt patients, in the training cohort. Baseline cohort of the figure: *NPM1*-mut. 18

Figure S5. Two-year overall survival comparison of gene co-mutations in *NPM1*-mut AML compared to ELN2022 risk classification in *NPM1*-wt patients, in the training cohort. Baseline cohort of the figure: *NPM1*-mut and *FLT3*-ITD. 18

Figure S6. Co-mutational landscape of patients with *NPM1*-mut and *FLT3*-ITD. 19

Figure S7. Co-mutational landscape of patients with *NPM1*-mut, *FLT3*-ITD and *DNMT3A*-mut. 19

Figure S8. Two-year overall survival comparison of gene co-mutations in *NPM1*-mut AML compared to ELN2022 risk classification in *NPM1*-wt patients, in the training cohort. Baseline cohort of the figure: *NPM1*-mut, *FLT3*-ITD and *DNMT3A*-wt. 20

Figure S9. Co-mutational landscape of patients with *NPM1*-mut, *FLT3*-ITD and *DNMT3A*-wt. 20

Figure S10. Overall survival of patients with *NPM1*-mut AML and *FLT3*-ITD according to HARMONY *NPM1*-mut classification. 21

Figure S11. Two-year overall survival comparison of gene co-mutations in *NPM1*-mut AML compared to ELN2022 risk classification in *NPM1*-wt patients, in the training cohort. Baseline cohort of the figure: *NPM1*-mut and absence of *FLT3*-ITD. 22

Figure S12. Co-mutational landscape of patients with *NPM1*-mut and absence of *FLT3*-ITD. 22

Figure S13. Two-year overall survival comparison of gene co-mutations in *NPM1*-mut AML compared to ELN2022 risk classification in *NPM1*-wt patients, in the training cohort. Baseline cohort of the figure: *NPM1*-mut, absence of *FLT3*-ITD and *DNMT3A*-mut. 23

Figure S15. Overall survival of patients with *NPM1*-mut AML and DNMT3A-*mut* according to HARMONY *NPM1*-mut classification. 24

Figure S16. Two-year overall survival comparison of gene co-mutations in *NPM1*-mut AML compared to ELN2022 risk classification in *NPM1*-wt patients, in the training cohort. Baseline cohort of the figure: *NPM1*-mut, absence of *FLT3*-ITD and *DNMT3A*-wt. 25

Figure S17. Co-mutational landscape of patients with *NPM1*-mut, absence of *FLT3*-ITD and *DNMT3A*-wt. 25

Figure S18. Overall survival of patients with *NPM1*-mut AML, absence of *FLT3*-ITD and *DNMT3A*-wt, stratified by *TET2* mutational status. 26

Figure S19. Two-year overall survival comparison of gene co-mutations in *NPM1*-mut AML compared to ELN2022 risk classification in *NPM1*-wt patients, in the training cohort. Baseline cohort of the figure: *NPM1*-mut, absence of *FLT3*-ITD, *DNMT3A*-wt and *TET2*-wt. 26

Figure S20. Comparative of overall survival according to *NPM1* classification (for *NPM1*-mut patients) and ELN2022 (for *NPM1*-wt patients), in the training cohort. 27

Figure S21. Overall survival according to *NPM1* classification, in training cohort (**A**, **B**), internal validation cohort (**C**, **D**) and external validation cohort (**E**, **F**), in patients aged ≤60 years (**A**, **C**, **E**) and >60 years at AML diagnosis (**B**, **D**, **F**). 28

Figure S22. *TP53* mutation impact on overall survival in all patients of the training cohort with *NPM1*-mut. 29

Figure S23. *RUNX1* mutation impact on overall survival in all patients of the training cohort with *NPM1*-mut (**A**) and in the subset with absence of *FLT3*-ITD (**B**). 30

Figure S24. *SRSF2* mutation impact on overall survival in the subset of patients of the training cohort with absence of *FLT3*-ITD (**A**) and in the subset with absence of *FLT3*-ITD and *DNMT3A*-wt (**B**). 30

Figure S25. *STAG2* and *RAD21* mutations impact on overall survival in the subset of the training cohort with absence of *FLT3*-ITD. 31

Figure S26. Overall survival of patients with *NPM1*-mut AML in the training cohort, stratified by cytogenetic aberrations, censoring patients that underwent allo-HSCT in CR1 at the transplant date (**A**) and without censoring transplanted patients (**B**). 31

Figure S27. Overall survival of *NPM1*-mut patients in training cohort. 32

Supplementary methods

NPM1-mut risk stratification

A multi-step analysis of clinically significant gene co-mutations associated to *NPM1*-mut was performed. At each step, combinations of up to two additional genes (either mutated or wildtype) that were present in at least 10 patients were explored. While late relapses in *NPM1*-mut AML have been reported, patient prognosis is mostly determined within the first 2 years from disease diagnosis in this AML subtype[1–3]. In fact, in HARMONY training cohort there was <10% difference between 2-year OS (62.6%) and 5-year OS (53.5%) and an increasing number of follow-up loses between those timepoints (**Figure S27**), so 2-year OS was selected for outcome comparison. The 2-year-OS for each combination was estimated using 100-fold bootstrap sampling and compared to the 2-year OS of *NPM1* wildtype (-wt) patients in the same dataset. Cloglog transformation of survival curves was used for testing differences at this fixed 2-year timepoint, as previously recommended[4]. Only combinations with statistically significant differences (cloglog p-value <0.05) compared to their respective opposites, in at least 60% of bootstrap sampling comparisons, were initially selected and represented in **Figures S4**, **S8**, **S11**, **S13**, **S16** and **S19**. Finally, gene mutation combinations that allowed patient reclassification into different European LeukemiaNet (ELN) 2022 risk categories were selected[5]. When multiple co-mutational patterns could potentially lead into patient reclassification, the most representative (i.e. with higher number of patients) was selected.

Exploratory analyses demonstrated similar findings with *IDH1*-mut and *IDH2*-mut in *NPM1*-mut (**Figures S1** and **S2**), so they were combined as *IDH*-mut (any mutated) or *IDH*-wt (both wildtype) in the final risk classification.

In the first step, combinations that met the aforementioned criteria are depicted in **Figure S1**. Baseline cohort (i.e. all *NPM1*-mut) presented a 2-year OS between ELN2022 favorable-risk and ELN2022 intermediate-risk for *NPM1*-wt in the same dataset. However, patients with *FLT3*-ITD

and *DNMT3A* had a 2-year OS that was different to ELN2022 intermediate-risk (cloglog p-value <0.001) and closer to ELN2022 adverse-risk patients (cloglog p-value 0.834), so this combination was selected. The deleterious effect of *DNMT3A*-mut was confirmed when the subset with *NPM1*-mut and *FLT3*-ITD was analyzed (**Figure S5**) and therefore triple-mutated patients (i.e. *NPM1*-mut, *FLT3*-ITD and *DNMT3A*-mut) were classified as "*NPM1* adverse". Subsequently, the subset with *NPM1*-mut, *FLT3*-ITD and *DNMT3A*-wt was evaluated (**Supplementary Figure S8**). This subset had a 2-year OS between ELN2022 favorable-risk and ELN2022 intermediate-risk, but *IDH*-mut patients had a 2-year OS that was distinct to ELN2022 intermediate-risk (cloglog p-value 0.013) and was closer to ELN2022 favorable-risk (cloglog p-value 0.409) and were therefore classified as "*NPM1* favorable". Conversely, *IDH*-wt patients had a 2-year OS that was different from ELN2022 favorable-risk (cloglog p-value <0.001) and closer to ELN2022 intermediate-risk (cloglog p-value 0.913) and were therefore classified as "*NPM1* intermediate". Similar methodology was applied to classify the remainder of NPM1-mut patients (**Supplementary Figures S11**, **S13** and **S16**).

References

1. Bertoli S, Tavitian S, Bérard E, et al: More than ten percent of relapses occur after five years in AML patients with NPM1 mutation. Leuk Lymphoma 61:1226–1229, 2020

2. Giupponi C, Bertoli D, Borlenghi E, et al: Myeloid neoplasm occurrence during stable molecular remission of NPM1-mutated AML: are we facing secondary disease or AML relapse? Blood Cancer J 13, 2023

3. Othman J, Potter N, Ivey A, et al: Molecular, clinical, and therapeutic determinants of outcome in NPM1-mutated AML. Blood 144:714–728, 2024

4. Klein JP, Logan B, Harhoff M, et al: Analyzing survival curves at a fixed point in time. Stat Med 26:4505–4519, 2007

5. Döhner H, Wei AH, Appelbaum FR, et al: Diagnosis and management of AML in adults: 2022 recommendations from an international expert panel on behalf of the ELN. Blood 140:1345–1377, 2022

Supplementary tables

Table S1. List of genes included in both NGS panels of the training cohort.

**Genes included in both NGS panels**
ASXL1
BCOR
CEBPA
DNMT3A
EZH2
FLT3
IDH1
IDH2
JAK2
KIT
KRAS
NPM1
NRAS
PTEN
PTPN11
RUNX1
SF3B1
SRSF2
STAG2
TET2
TP53
U2AF1
WT1
ZRSR2

Table S2. *IDH1* and *IDH2* mutation variants in the training cohort.

	***IDH1* R132H**	***IDH1* R132C**	**Other *IDH1* mutations**	***IDH2* R140Q**	***IDH2* R172K**	**Other *IDH2* mutations**
**Number of patients**	45	9	4	58	1	1

* Information regarding *IDH1* and *IDH2* variants was provided for 118 patients with *IDH* mutations in the training cohort

Table S3. Baseline characteristics training cohort, comparing patients with *NPM1* mutation (*NPM1*-mut) to patients with *NPM1* wildtype (*NPM1*-wt).

	***NPM1*-mut training cohort (n=1001)**	***NPM1*-wt training cohort (n=2473)**	**p-value**
**Female sex**	543 (54.2%)	1016 (41.1%)	<0.001
**Median age in years (range)**	52.9 (18 - 81)	55 [18 - 84.5]	0.025
Age ≥60 years	269 (26.8%)	763 (30.9%)	0.022
**AML type**			
De novo AML	963 (96.2%)	2198 (88.9%)	<0.001
Secondary AML	38 (3.8%)	275 (11.1%)	<0.001
Prior HM	15 (1.5%)	156 (6.3%)	
Therapy-related AML	23 (2.3%)	119 (4.8%)	
**Hemoglobin (g/dL)**	8.9 [Q1 = 7.6, Q3 = 10.3]	9.1 [Q1 = 7.8, Q3 = 10.3]	0.3062
**WBC (x10^9/L)**	23.8 [Q1 = 6.9, Q3 = 62.8]	6.2 [Q1 = 2.2, Q3 = 27.2]	<0.001
(WBC> 100 x10^9/L)	136 (13.6%)	140 (5.7%)	<0.001
**Platelets (x10^9/L)**	66.5 [Q1 = 38, Q3 = 116]	57 [Q1 = 31, Q3 = 108]	<0.001
**Bone marrow % of blasts**	75 [Q1 = 45, Q3 = 89]	55 [Q1 = 30, Q3 = 80]	<0.001
**ELN 2022**			<0.001
Favorable	601 (60%)	335 (15%)	
Intermediate	391 (39.1%)	578 (26%)	
Adverse	9 (0.9%)	1332 (59%)	
***FLT3*-ITD**	393 (39.3%)	316 (13%)	<0.001
**Treatment response**			
CRc	874 (87.3%)	2035 (82.3%)	<0.001
Refractory	89 (8.9%)	342 (13.8%)	<0.001
Not evaluable	38 (3.8%)	96 (3.9%)	1
**Early death**			
30-day mortality	38 (3.8%)	96 (3.9%)	1
60-day mortality	62 (6.2%)	226 (9.1%)	0.004
**Allogeneic HSCT**	341 (34.1%)	1130 (45.7%)	<0.001
In CR1	242 (24.2%)	998 (40%)	<0.001
In other situations	99 (9.9%)	132 (5.7%)	<0.001
**Median survival in years (95% CI)**	8.25 (5.14-9.77)	1.83 (1.69-2.05)	<0.001

Table S4. Comparison of HARMONY *NPM1* and ELN2022 risk classification criteria for *NPM1*-mut AML.

**HARMONY *NPM1* risk classification**	**Combination of gene mutations**	**ELN2022 risk classification**
NPM1 favorable	• Absence *FLT3*-ITD<br>• *DNMT3A*-wt<br>• *TET2*-wt	Favorable
	• Absence *FLT3*-ITD<br>• *DNMT3A*-mut<br>• *TET2*-wt	Favorable
	• *FLT3*-ITD<br>• *DNMT3A*-wt<br>• *IDH*-mut	Intermediate*
NPM1 intermediate	• Absence *FLT3*-ITD<br>• *DNMT3A*-wt<br>• *TET2*-mut	Favorable*
	• Absence *FLT3*-ITD<br>• *DNMT3A*-mut<br>• *IDH*-mut	Favorable*
	• *FLT3*-ITD<br>• *DNMT3A*-wt<br>• *IDH*-wt	Intermediate
NPM1 adverse	• *FLT3*-ITD<br>• *DNMT3A*-mut	Intermediate*

* Indicates change in risk category from ELN2022 to HARMONY *NPM1* classification

Table S5. Predictive performance of 5-year OS of HARMONY *NPM1* risk classification compared to ELN2022, measured by the time-dependent receiver operating curve (AUC(t))

	**HARMONY *NPM1* classification**	**ELN2022**
**Training cohort**	0.695	0.635
**Internal validation cohort**	0.643	0.632
**External validation cohort**	0.600	0.558

Table S6. Univariable and multivariable analysis of overall survival in the training cohort, including allogeneic stem cell transplantation in first complete remission (allo-HSCT in CR1) as a time-dependent covariate.

	**Univariable**		**Multivariable**	
	**HR (95% CI)**	**p-value**	**HR (95% CI)**	**p-value**
**All patients (n=951)**				
Age >60 years	2 (1.6-2.5)	<0.001	1.74 (1.42-2.15)	<0.001
WBC >100 (x10^9/L)	2.2 (1.7-2.9)	<0.001	1.40 (1.08-1.80)	0.011
Prior HM*	2.9 (1.38-6.2)	0.005	3.42 (1.82-6.44)	<0.001
Therapy-related AML*	1.2 (0.59-2.6)	0.573	1.52 (0.86-2.71)	0.153
NPM1 Intermediate**	1.8 (1.4-2.4)	<0.001	1.90 (1.49-2.43)	<0.001
NPM1 Adverse**	3.2 (2.5-4.1)	<0.001	3.32 (2.35-4.68)	<0.001
ELN2022 (Intermediate/Adverse vs Favorable)	2 (1.6-2.4)	<0.001	1.01 (0.75-1.35)	0.964
Allo-HSCT in CR1	0.9 (0.86-0.94)	<0.001	0.84 (0.81-0.89)	<0.001
***NPM1* Favorable (n=496)**				
Age >60 years	2.3 (1.7-3.2)	<0.001	2.39 (1.73-3.30)	<0.001
WBC >100 (x10^9/L)	1.9 (1.1-3.3)	0.025	1.80 (1.07-3.03)	0.027
ELN2022 (Intermediate/Adverse vs Favorable)	0.87 (0.48-1.6)	0.646	0.85 (0.50-1.43)	0.536
Allo-HSCT in CR1	0.88 (0.82-0.94)	<0.001	0.88 (0.82-0.94)	<0.001
***NPM1* Intermediate (n=230)**				
Age >60 years	1.8 (1.2-2.7)	0.007	1.70 (1.12-2.46)	0.011
WBC >100 (x10^9/L)	1.5 (0.94-2.5)	0.084	1.40 (0.87-2.21)	0.174
ELN2022 (Intermediate/Adverse vs Favorable)	1.1 (0.72-1.6)	0.724	1.00 (0.71-1.49)	0.891
Allo-HSCT in CR1	0.9 (0.83-0.97)	0.009	0.9 (0.83-0.98)	0.017
***NPM1* Adverse (n=225)**				
Age >60 years	1.8 (1.2-2.7)	0.009	1.26 (0.86-1.85)	0.23
WBC >100 (x10^9/L)	1.7 (1.1-2.7)	0.012	1.26 (0.87-1.84)	0.22
Allo-HSCT in CR1	0.66 (0.57-0.76)	<0.001	0.66 (0.57-0.77)	<0.001

* De novo AML was used as reference

** *NPM1* Favorable was used as reference

Table S7. Univariable and multivariable analysis of overall survival in the internal validation cohort, including allogeneic stem cell transplantation in first complete remission (allo-HSCT in CR1) as a time-dependent covariate.

	**Univariable**		**Multivariable**	
	**HR (95% CI)**	**p-value**	**HR (95% CI)**	**p-value**
**All patients (n=620)**				
Age >60 years	1.7 (1.3-2.1)	<0.001	1.53 (1.22-1.92)	<0.001
WBC >100 (x10^9/L)	2.1 (1.6-2.7)	<0.001	1.61 (1.25-2.08)	<0.001
Prior HM*	1 (0.67-1.6)	0.861	1.23 (0.83-1.83)	0.302
Therapy-related AML*	1.4 (0.64-3.2)	0.38	0.99 (0.41-2.42)	0.987
NPM1 Intermediate**	1.5 (1.1-2)	0.005	1.14 (0.85-1.53)	0.365
NPM1 Adverse**	2.6 (2-3.3)	<0.001	1.53 (1.07-2.19)	0.021
ELN2022 (Intermediate/Adverse vs Favorable)	2.1 (1.7-2.7)	<0.001	1.49 (1.09-2.03)	0.013
Allo-HSCT in CR1	0.91 (0.87-0.95)	<0.001	0.89 (0.85-0.93)	<0.001
***NPM1* Favorable (n=267)**				
Age >60 years	2.1 (1.4-3)	<0.001	1.96 (1.35-2.80)	<0.001
WBC >100 (x10^9/L)	1.8 (1.1-3.1)	0.027	1.57 (0.93-2.60)	0.094
ELN2022 (Intermediate/Adverse vs Favorable)	1.4 (0.83-2.3)	0.21	1.18 (0.72-1.90)	0.508
Allo-HSCT in CR1	0.99 (0.94-1)	0.635	0.99 (0.94-1.00)	0.605
***NPM1* Intermediate (n=176)**				
Age >60 years	1.5 (0.97-2.3)	0.07	1.24 (0.81-1.90)	0.322
WBC >100 (x10^9/L)	2.3 (1.4-3.6)	<0.001	1.72 (1.09-2.72)	0.02
ELN2022 (Intermediate/Adverse vs Favorable)	1.5 (0.97-2.3)	0.066	1.56 (1.02-2.38)	0.041
Allo-HSCT in CR1	0.85 (0.76-0.94)	0.002	0.84 (0.75-0.94)	0.003
***NPM1* Adverse (n=177)**				
Age >60 years	1.7 (1.1-2.5)	0.01	1.42 (0.96-2.10)	0.082
WBC >100 (x10^9/L)	1.5 (0.99-2.2)	0.055	1.57 (1.08-2.30)	0.019
Allo-HSCT in CR1	0.79 (0.71-0.88)	<0.001	0.78 (0.69-0.88)	<0.001

* De novo AML was used as reference

** *NPM1* Favorable was used as reference

Table S8. Comparison of baseline patient characteristics between training cohort and external validation cohort.

	Training cohort (n=1001)	External validation cohort (n=585)	p-value
**Female sex**	543 (54.2%)	322 (55%)	0.7985
**Median age in years (range)**	52.9 (18 - 81)	56.4 (16 -79)	<0.0001
Age ≥60 years	269 (26.8%)	224 (38.3%)	<0.0001
**AML type**			
De novo AML	963 (96.2%)	551 (94.2%)	0.0628
Secondary AML	38 (3.8%)	34 (5.8%)	
Prior HM	15 (1.5%)	28 (4.8%)	0.0001
Therapy-related	23 (2.3%)	6 (1%)	0.0681
**Hemoglobin (g/dL)**	8.9 [Q1 = 7.6, Q3 = 10.3]	9.3 [Q1 = 7.8, Q3 = 10.9]	0.01248
**WBC (x10^9/L)**	23.8 [Q1 = 6.9, Q3 = 62.8]	32.6 [Q1 = 12.2, Q3 = 74.5]	<0.0001
(WBC> 100 x10^9/L)	136 (13.6%)	103 (17.6%)	0.0308
**Platelets (x10^9/L)**	66.5 [Q1 = 38, Q3 = 116]	63 [Q1 = 38, Q3 = 106]	0.2769
**BM % of blasts**	75 [Q1 = 45, Q3 = 89]	72 [Q1 = 50, Q3 = 89]	0.7606
**ELN 2022**			
Favorable	601 (60%)	340 (58.1%)	
Intermediate	391 (39.1%)	241 (41.2%)	0.4018
Adverse	9 (0.9%)	4 (0.7%)	
***FLT3*-ITD**	393 (39.3%)	243 (41.5%)	0.401
**Treatment response**			
CRc	874 (87.3%)	524 (89.6%)	
Refractory	89 (8.9%)	26 (4.4%)	0.0002
Not evaluable	38 (3.8%)	35 (6%)	
**Early death**			
30-day mortality	38 (3.8%)	35 (6%)	0.0449
60-day mortality	62 (6.2%)	40 (6.8%)	0.6143
**Allogeneic HSCT**	341 (34.1%)	157 (41.7%)*	0.0028
In CR1	242 (24.2%)	NA	
In other situations	99 (9.9%)	NA	
**Median survival in years (95% CI)**	8.25 (5.14-9.77)	4.48 (3.2 - 9.72)	<0.0001

* Information regarding allo-HSCT was provided for 378 patients in the external validation cohort

Table S9. Comparison of baseline patient characteristics between internal validation cohort and external validation cohort.

	Internal validation cohort (n=762)	External validation cohort (n=585)	p-value
**Female sex**	419 (55%)	322 (55%)	1
**Median age in years (range)**	57 (18 - 86)	56.4 (16 -79)	0.0933
Age ≥60 years	320 (42%)	224 (38.3%)	0.1877
**AML type**			
De novo AML	573 (90.5%)	551 (94.2%)	0.0166
Secondary AML	60 (9.5%)	34 (5.8%)	
Prior HM	48 (7.6%)	28 (4.8%)	0.2331
Therapy-related	12 (1.6%)	6 (1%)	0.3843
**Hemoglobin (g/dL)**	9.2 [Q1 = 8.2, Q3 = 10.5]	9.3 [Q1 = 7.8, Q3 = 10.9]	0.8477
**WBC (x10^9/L)**	36.8 [Q1 = 13.4, Q3 = 85.6]	32.6 [Q1 = 12.2, Q3 = 74.5]	0.154
(WBC> 100 x10^9/L)	144 (20.4%)	103 (17.6%)	0.5441
**Platelets (x10^9/L)**	66 .5 [Q1 = 38.2, Q3 = 111]	63 [Q1 = 38, Q3 = 106]	0.373
**BM % of blasts**	72.75 [Q1 = 52, Q3 = 87]	72 [Q1 = 50, Q3 = 89]	0.8679
**ELN 2022**			
Favorable	405 (53.2%)	340 (58.1%)	
Intermediate	346 (45.4%)	241 (41.2%)	0.0886
Adverse	11 (1.4%)	4 (0.7%)	
***FLT3*-ITD**	349 (45.8%)	243 (41.5%)	0.1319
**Treatment response**			
CRc	606 (79.5%)	524 (89.6%)	
Refractory	95 (12.5%)	26 (4.4%)	<0.0001
Not evaluable	61 (8%)	35 (6%)	
**Early death**			
30-day mortality	61 (8%)	35 (6%)	0.1528
60-day mortality	88 (11.5%)	40 (6.8%)	0.0035
**Allogeneic HSCT**	215 (29.3%)	157 (41.7%)	0.0151
In CR1	130 (60.5%)	NA	
In other situations	85 (39.5%)	NA	
**Median survival in years (95% CI)**	2.84 (2.06 - 4.09)	4.48 (3.2 - 9.72)	<0.0001

Supplementary figures

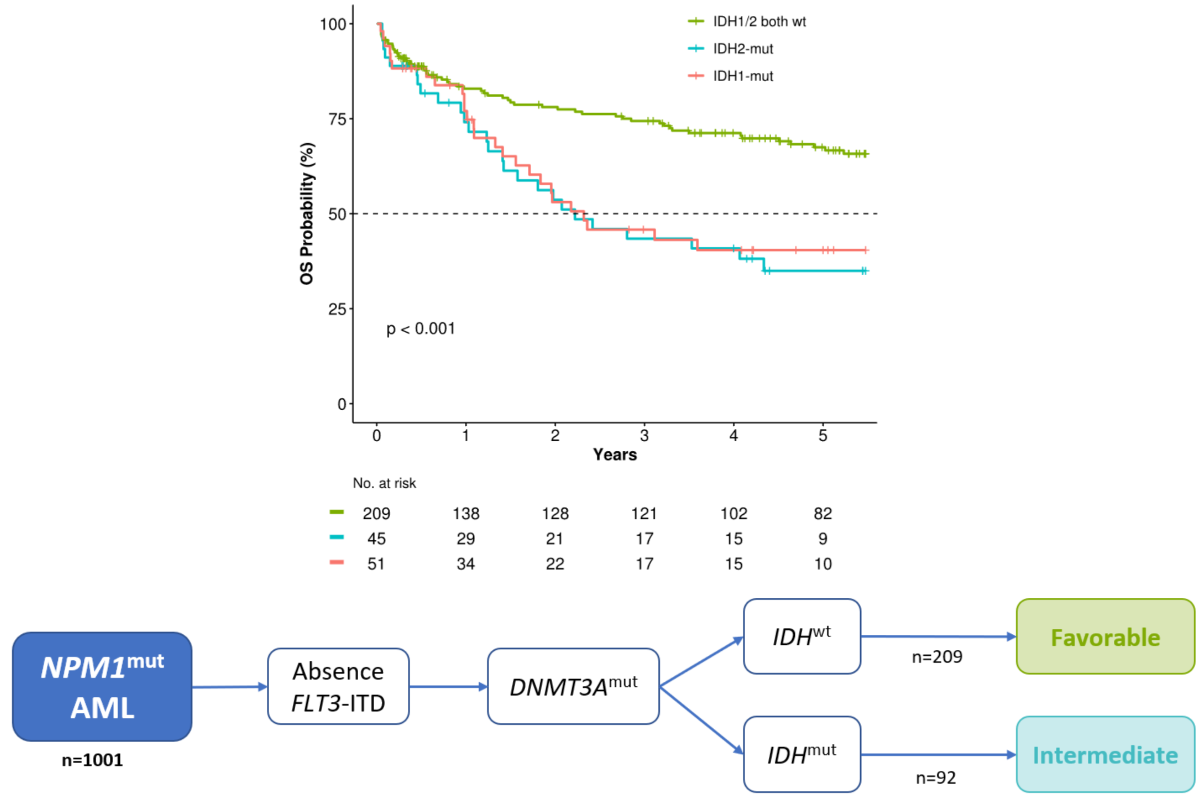

Figure S1. *IDH1/2* sub-analysis of overall survival in patients with *NPM1*-mut, absence *FLT3*-ITD and *DNMT3A*-mut.

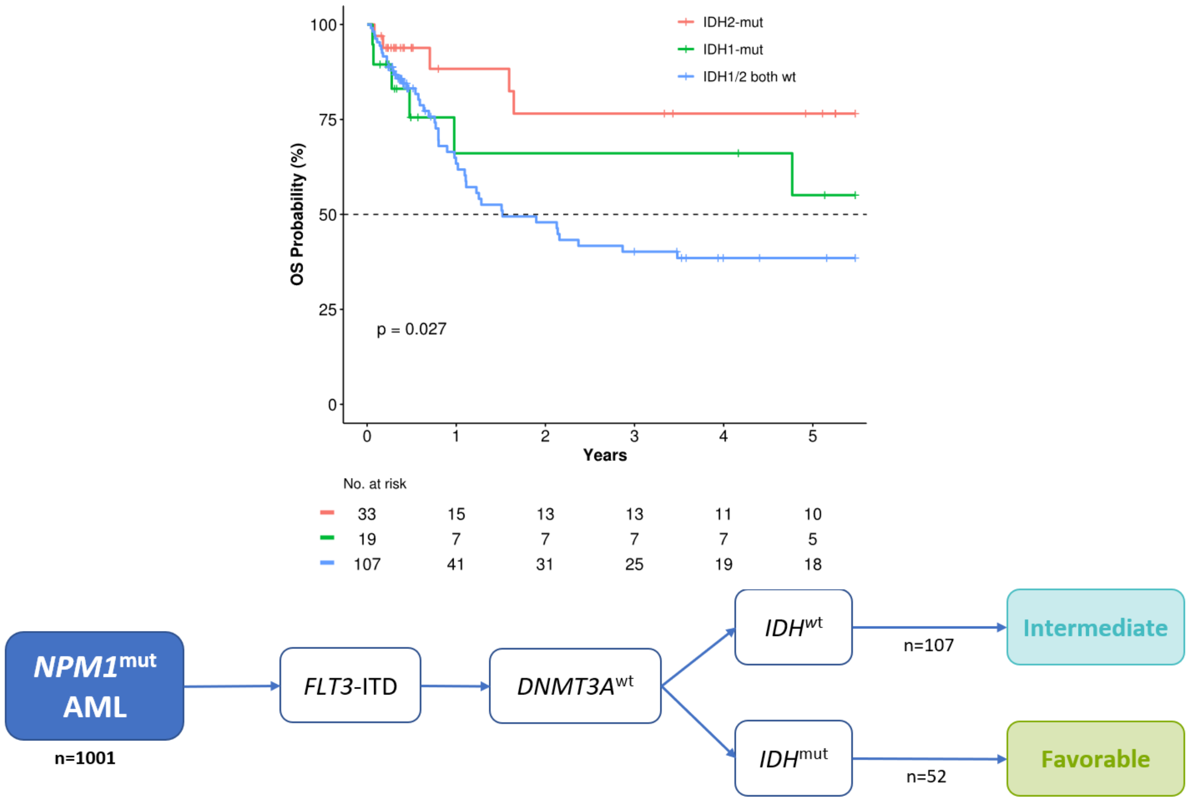

Figure S2. *IDH1/2* sub-analysis of overall survival in patients with *NPM1*-mut, *FLT3*-ITD and *DNMT3A*-wt.

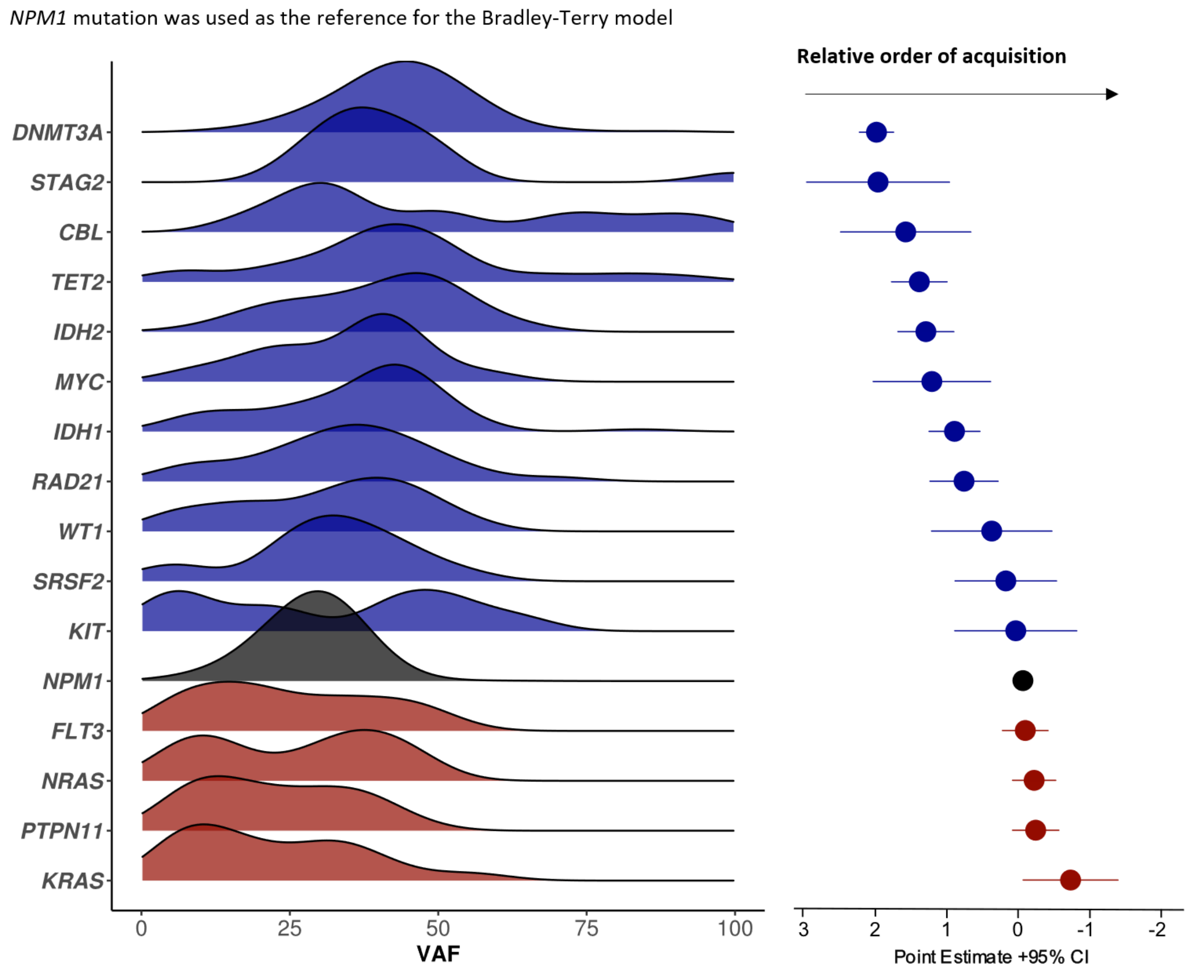

Figure S3. Variant allele frequency of most-frequently mutated genes in *NPM1*-mut AML (training cohort) and Bradley-Terry model of relative order of mutation acquisition.

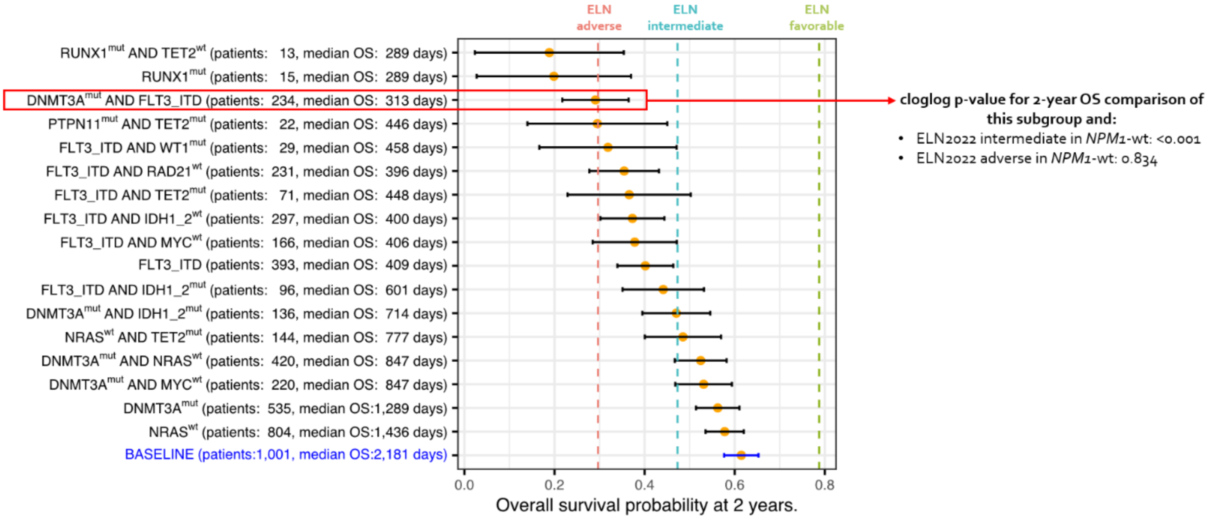

Figure S4. Two-year overall survival comparison of gene co-mutations in *NPM1*-mut AML compared to ELN2022 risk classification in *NPM1*-wt patients, in the training cohort. Baseline cohort of the figure: *NPM1*-mut.

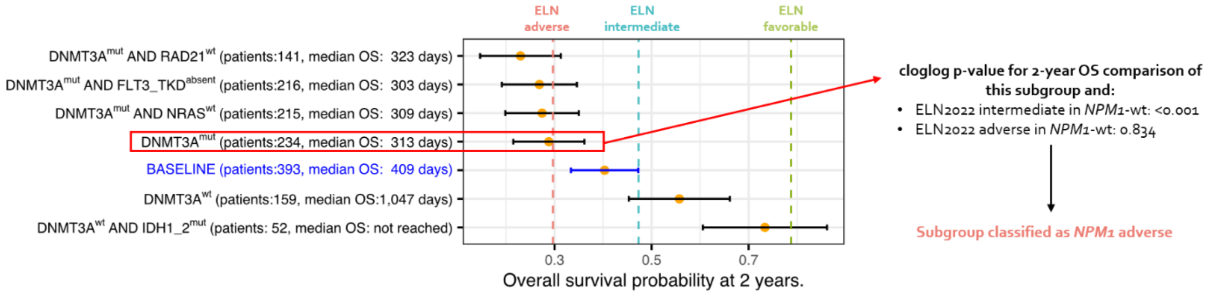

Figure S5. Two-year overall survival comparison of gene co-mutations in *NPM1*-mut AML compared to ELN2022 risk classification in *NPM1*-wt patients, in the training cohort. Baseline cohort of the figure: *NPM1*-mut and *FLT3*-ITD.

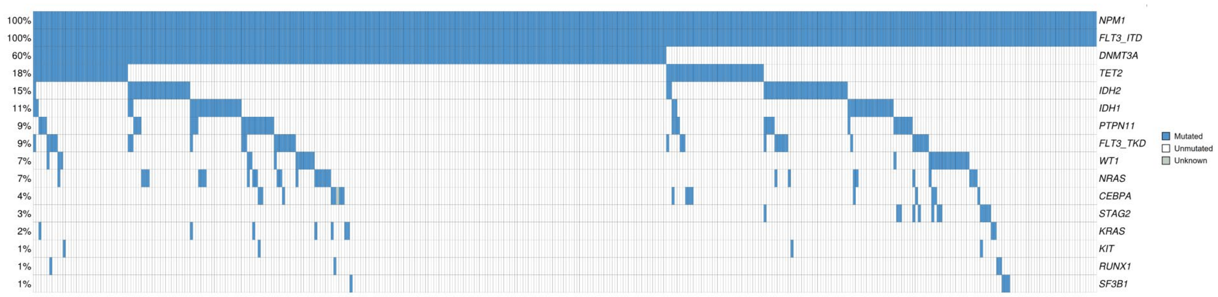

Figure S6. Co-mutational landscape of patients with *NPM1*-mut and *FLT3*-ITD.

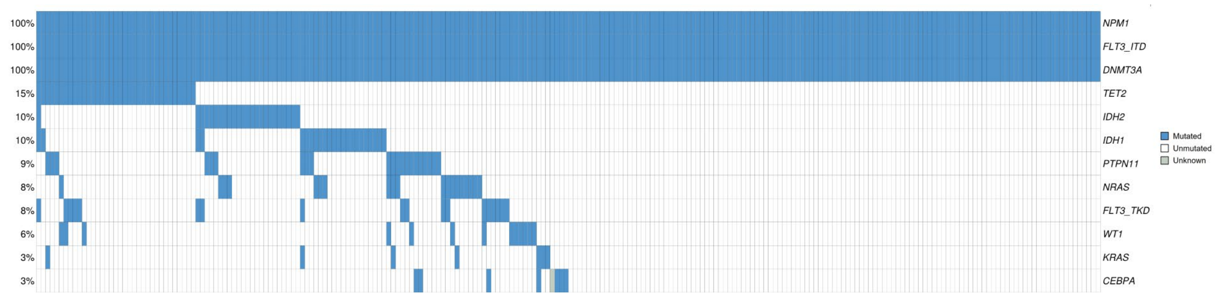

Figure S7. Co-mutational landscape of patients with *NPM1*-mut, *FLT3*-ITD and *DNMT3A*-mut.

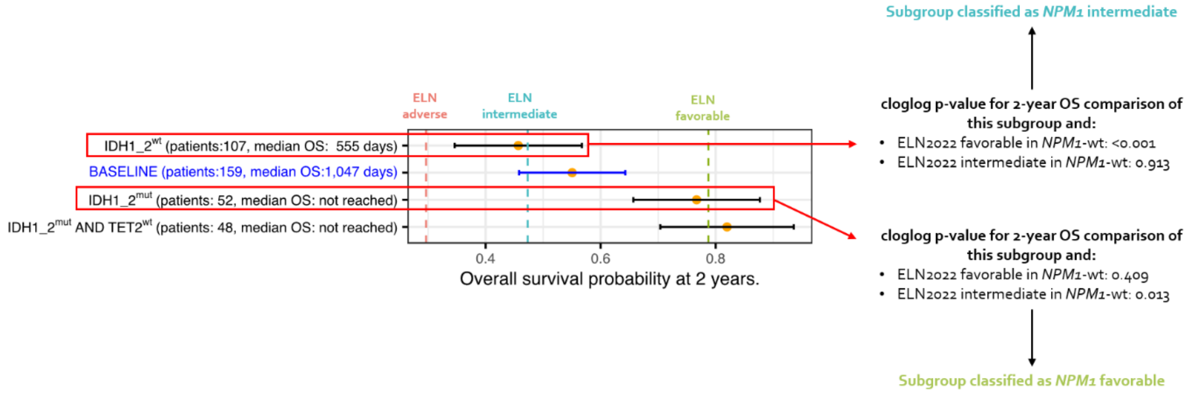

Figure S8. Two-year overall survival comparison of gene co-mutations in *NPM1*-mut AML compared to ELN2022 risk classification in *NPM1*-wt patients, in the training cohort. Baseline cohort of the figure: *NPM1*-mut, *FLT3*-ITD and *DNMT3A*-wt.

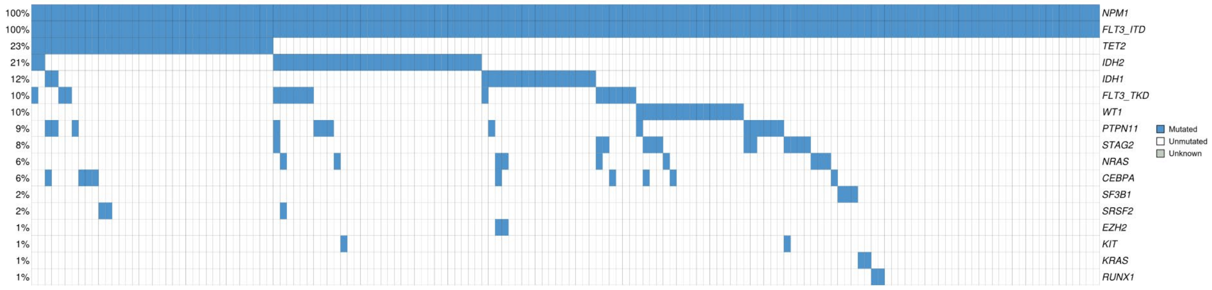

Figure S9. Co-mutational landscape of patients with *NPM1*-mut, *FLT3*-ITD and *DNMT3A*-wt.

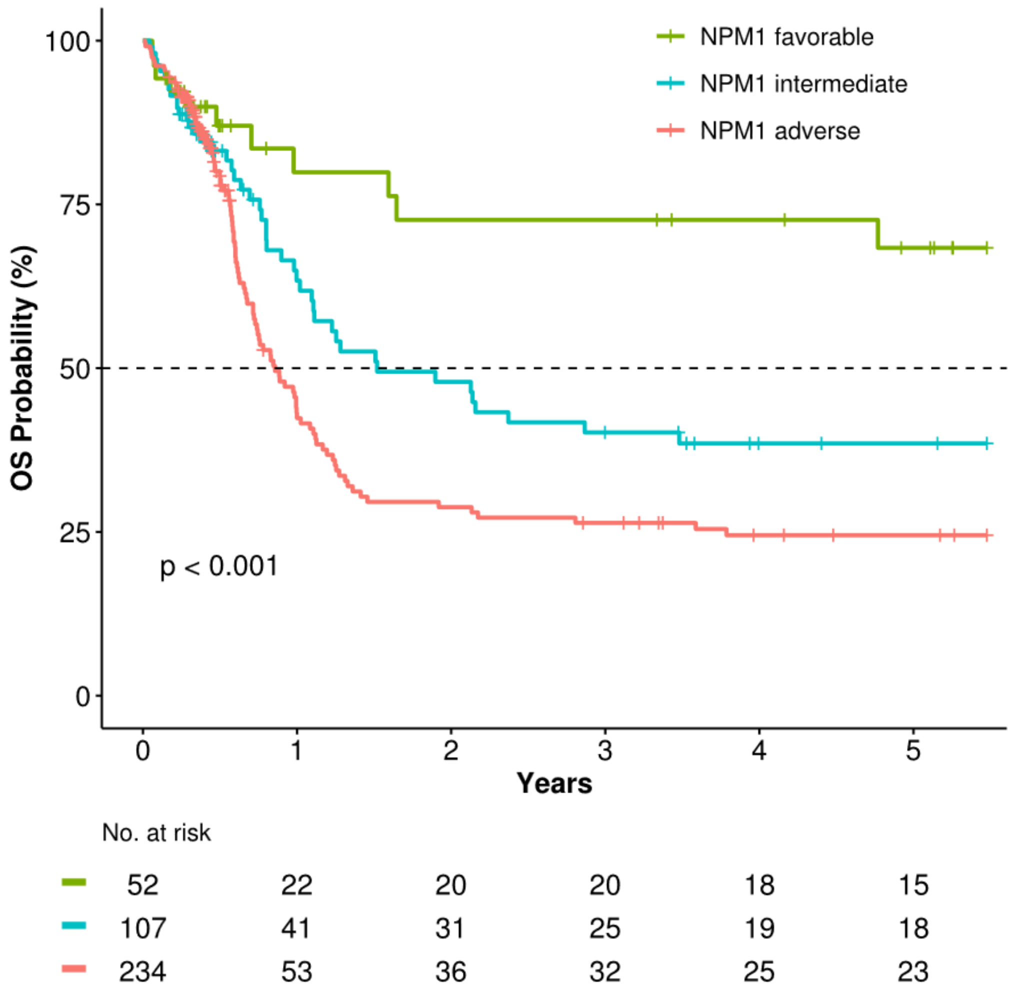

Figure S10. Overall survival of patients with *NPM1*-mut AML and *FLT3*-ITD according to HARMONY *NPM1*-mut classification.

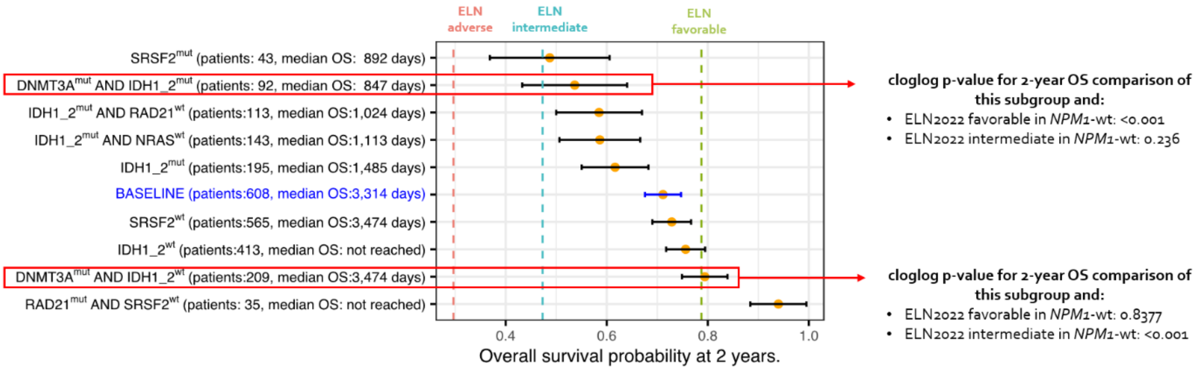

Figure S11. Two-year overall survival comparison of gene co-mutations in *NPM1*-mut AML compared to ELN2022 risk classification in *NPM1*-wt patients, in the training cohort. Baseline cohort of the figure: *NPM1*-mut and absence of *FLT3*-ITD.

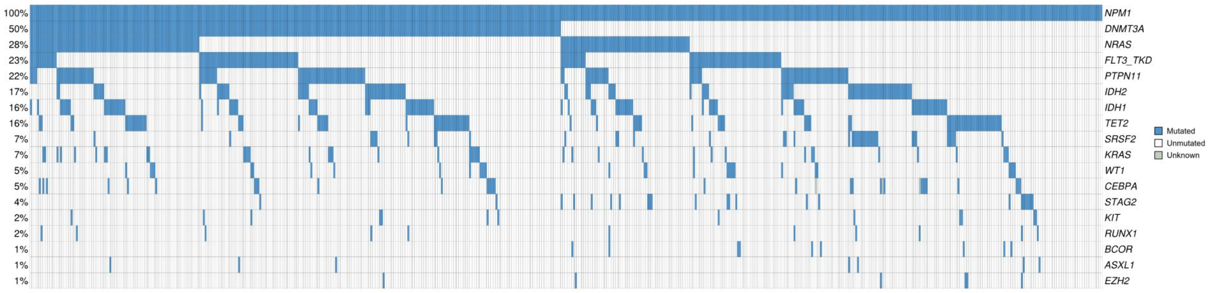

Figure S12. Co-mutational landscape of patients with *NPM1*-mut and absence of *FLT3*-ITD.

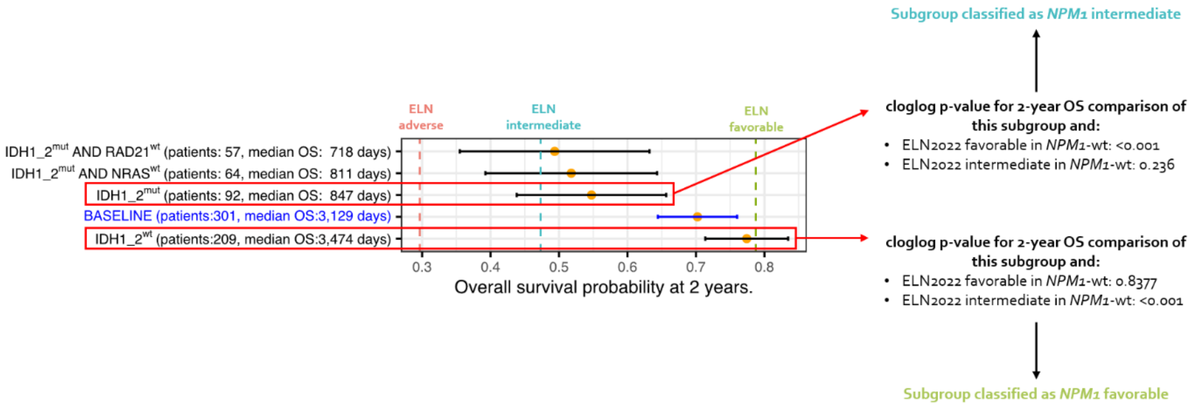

Figure S13. Two-year overall survival comparison of gene co-mutations in *NPM1*-mut AML compared to ELN2022 risk classification in *NPM1*-wt patients, in the training cohort. Baseline cohort of the figure: *NPM1*-mut, absence of *FLT3*-ITD and *DNMT3A*-mut.

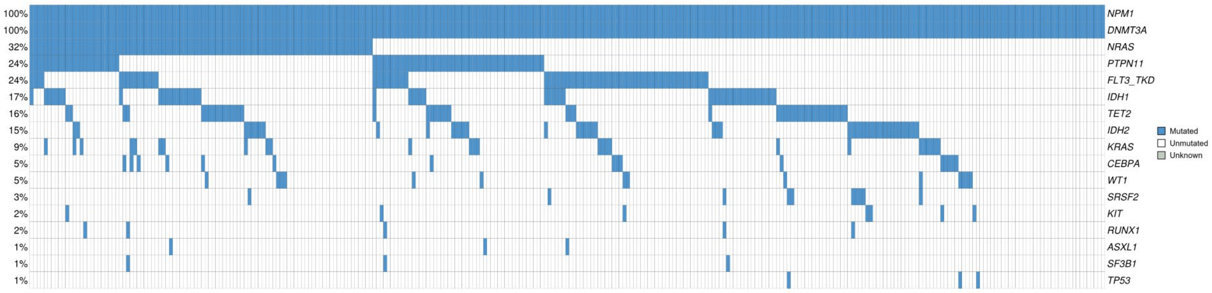

Figure S14. Co-mutational landscape of patients with *NPM1*-mut, absence of *FLT3*-ITD and *DNMT3A*-mut.

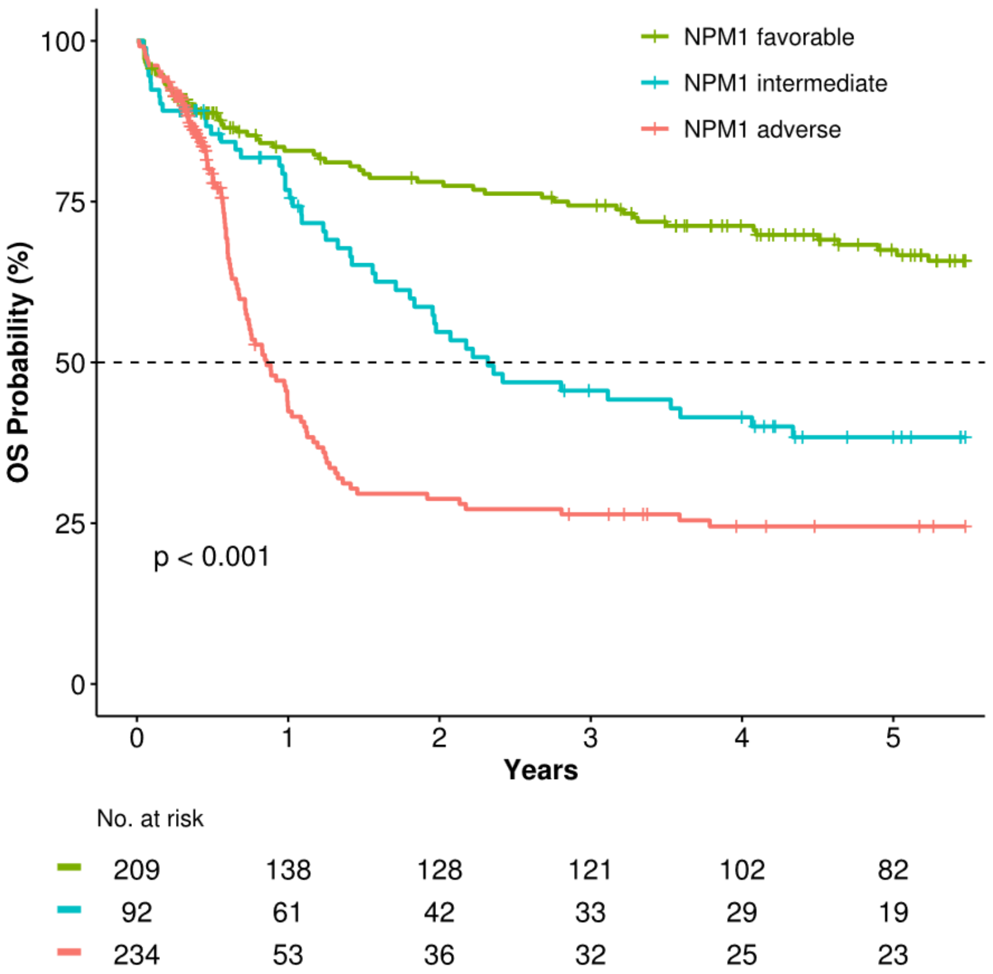

Figure S15. Overall survival of patients with *NPM1*-mut AML and DNMT3A-*mut* according to HARMONY *NPM1*-mut classification.

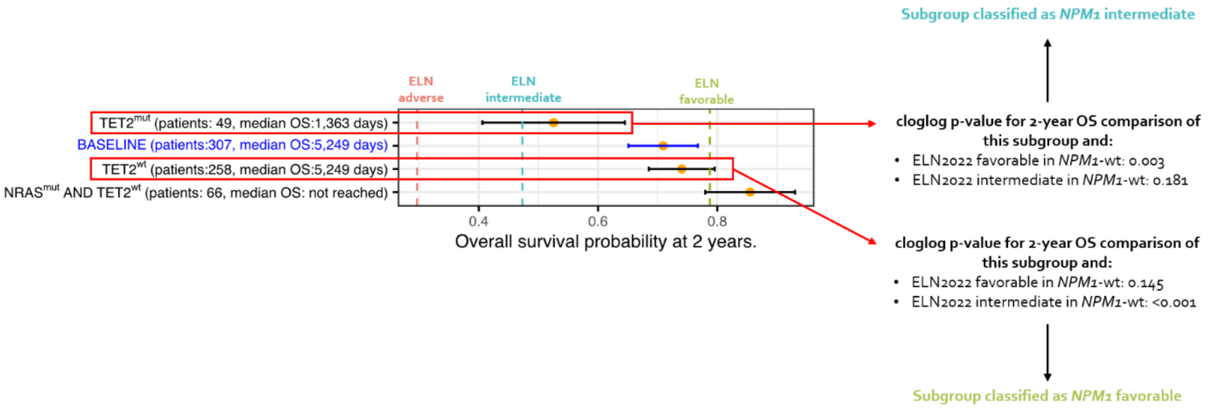

Figure S16. Two-year overall survival comparison of gene co-mutations in *NPM1*-mut AML compared to ELN2022 risk classification in *NPM1*-wt patients, in the training cohort. Baseline cohort of the figure: *NPM1*-mut, absence of *FLT3*-ITD and *DNMT3A*-wt.

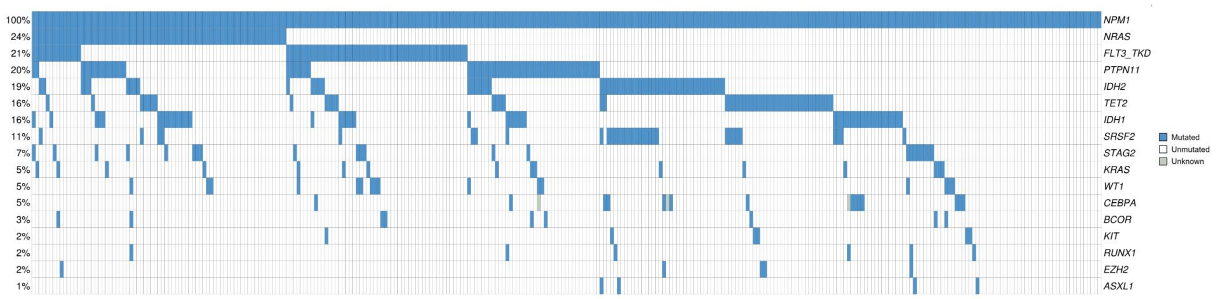

Figure S17. Co-mutational landscape of patients with *NPM1*-mut, absence of *FLT3*-ITD and *DNMT3A*-wt.

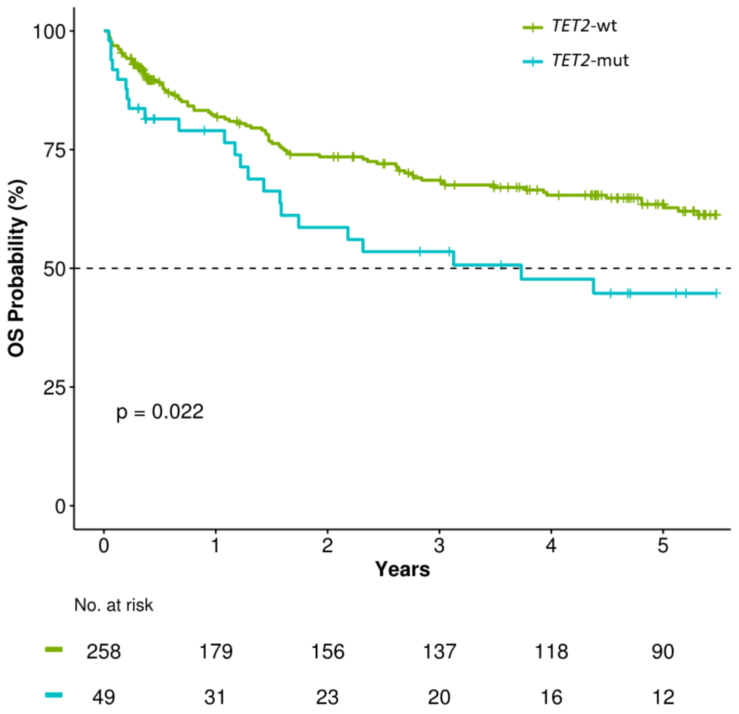

Figure S18. Overall survival of patients with *NPM1*-mut AML, absence of *FLT3*-ITD and *DNMT3A*-wt, stratified by *TET2* mutational status.

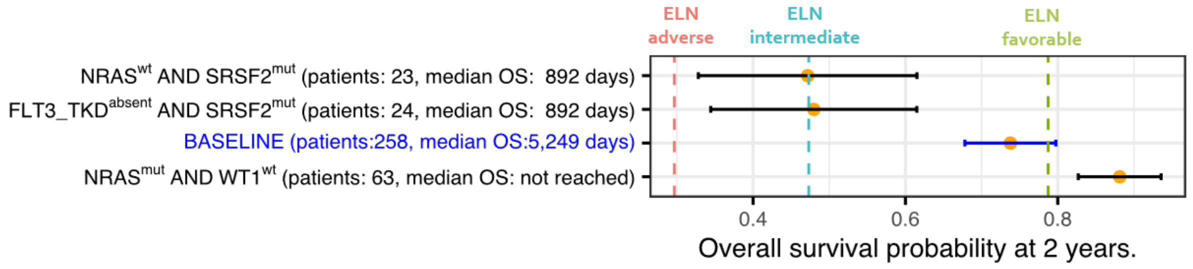

Figure S19. Two-year overall survival comparison of gene co-mutations in *NPM1*-mut AML compared to ELN2022 risk classification in *NPM1*-wt patients, in the training cohort. Baseline cohort of the figure: *NPM1*-mut, absence of *FLT3*-ITD, *DNMT3A*-wt and *TET2*-wt.

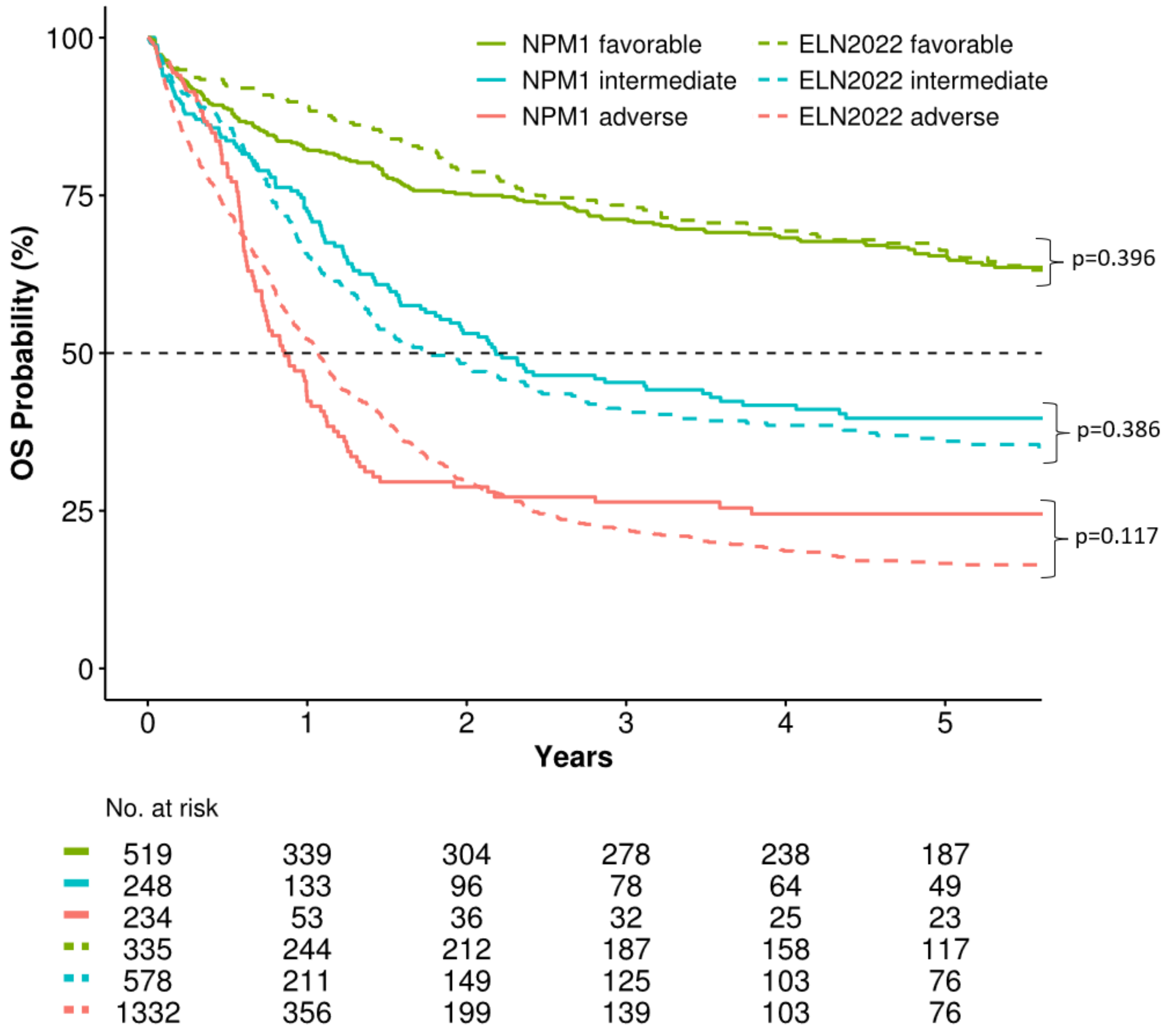

Figure S20. Comparative of overall survival according to *NPM1* classification (for *NPM1*-mut patients) and ELN2022 (for *NPM1*-wt patients), in the training cohort.

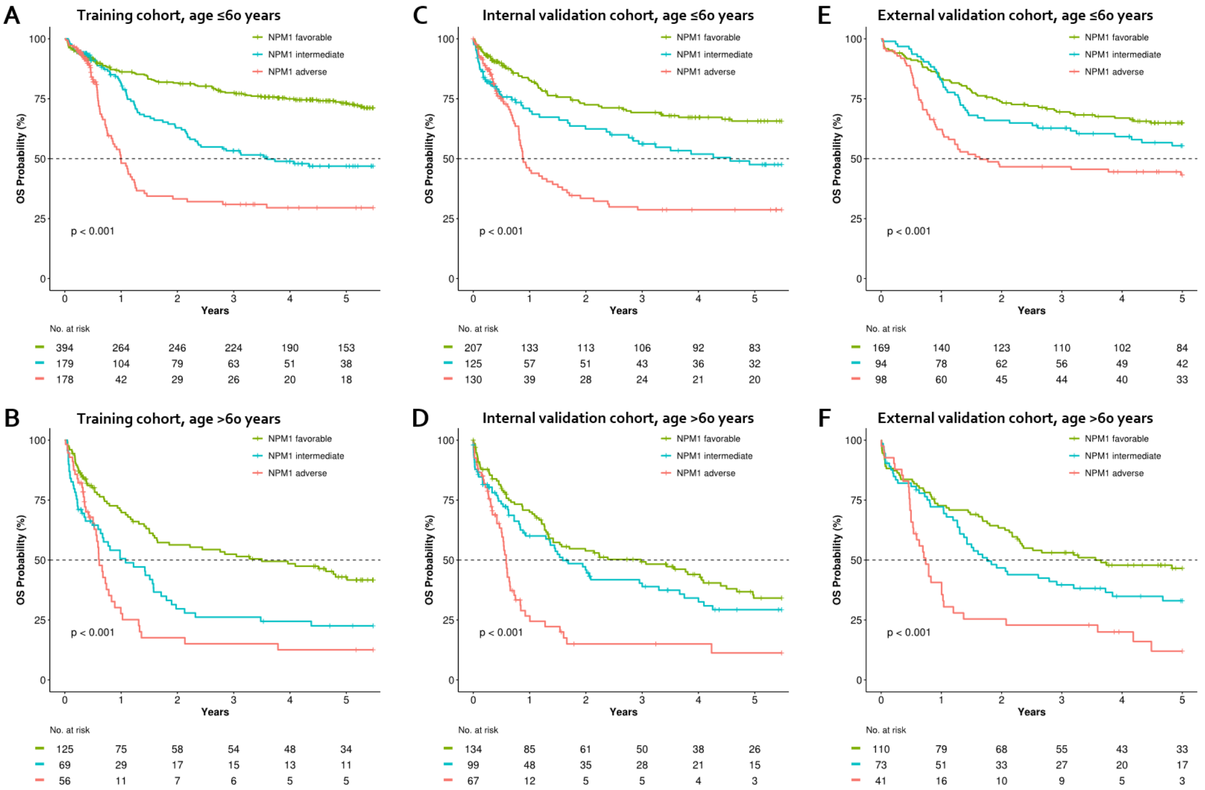

Figure S21. Overall survival according to *NPM1* classification, in training cohort (**A**, **B**), internal validation cohort (**C**, **D**) and external validation cohort (**E**, **F**), in patients aged ≤60 years (**A**, **C**, **E**) and >60 years at AML diagnosis (**B**, **D**, **F**).

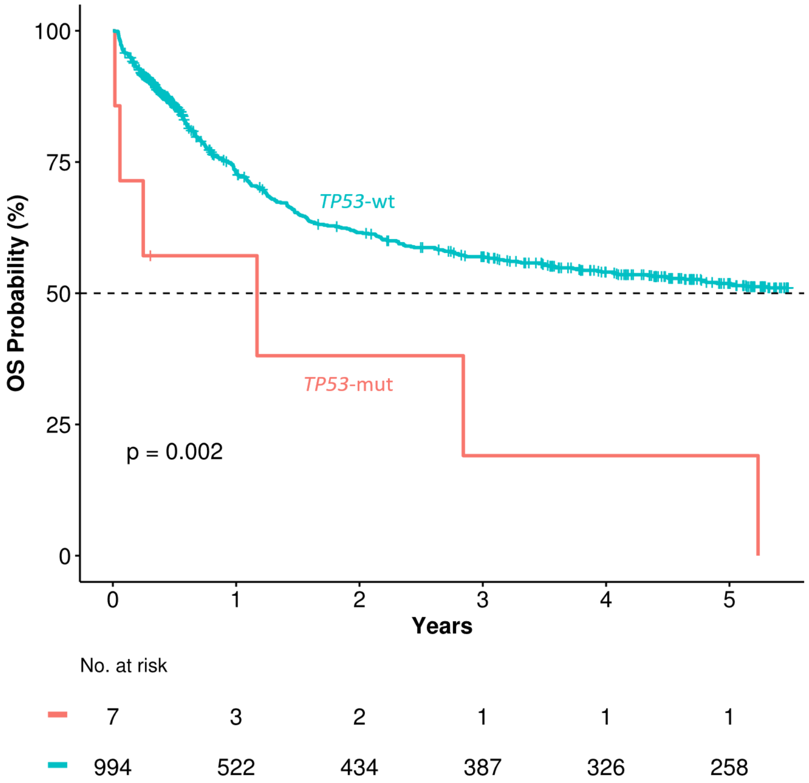

Figure S22. *TP53* mutation impact on overall survival in all patients of the training cohort with *NPM1*-mut.

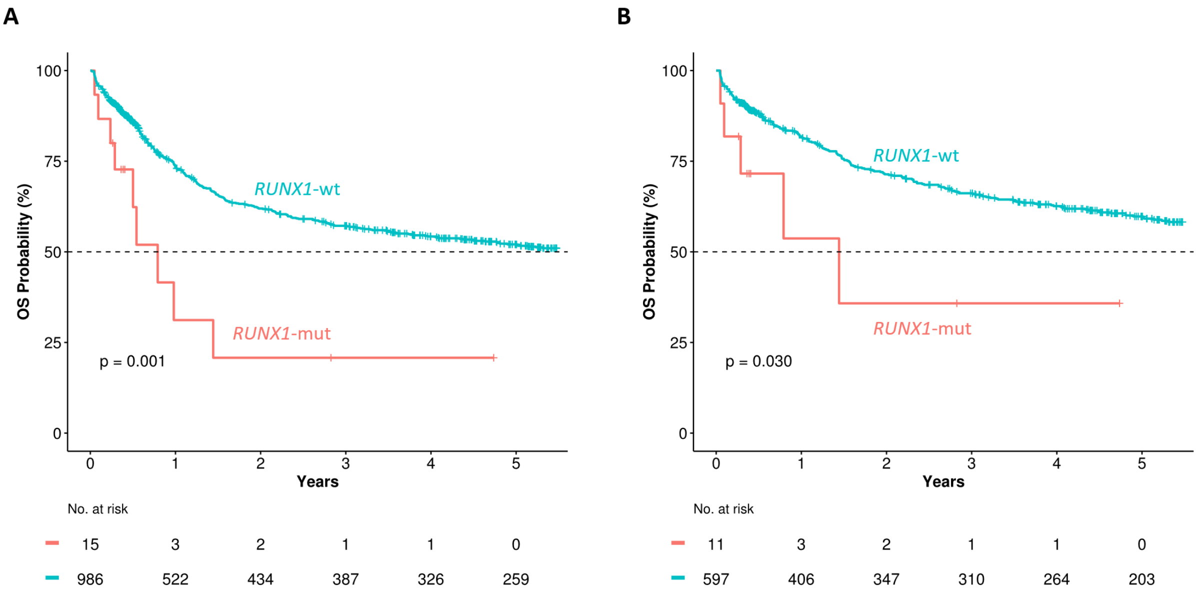

Figure S23. *RUNX1* mutation impact on overall survival in all patients of the training cohort with *NPM1*-mut (**A**) and in the subset with absence of *FLT3*-ITD (**B**).

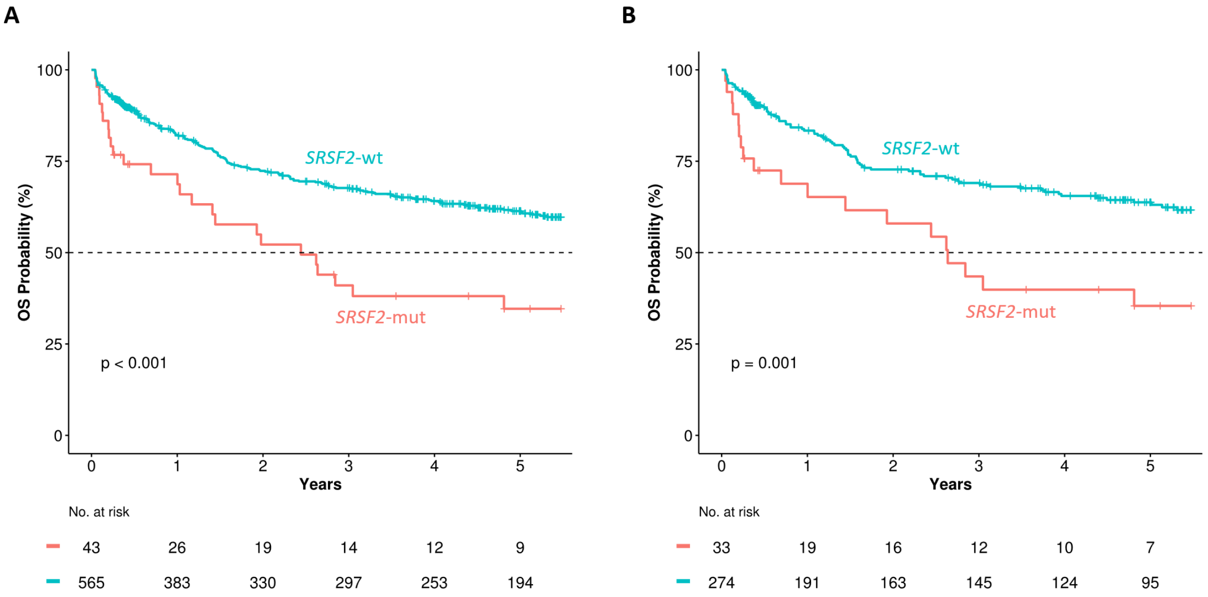

Figure S24. *SRSF2* mutation impact on overall survival in the subset of patients of the training cohort with absence of *FLT3*-ITD (**A**) and in the subset with absence of *FLT3*-ITD and *DNMT3A*-wt (**B**).

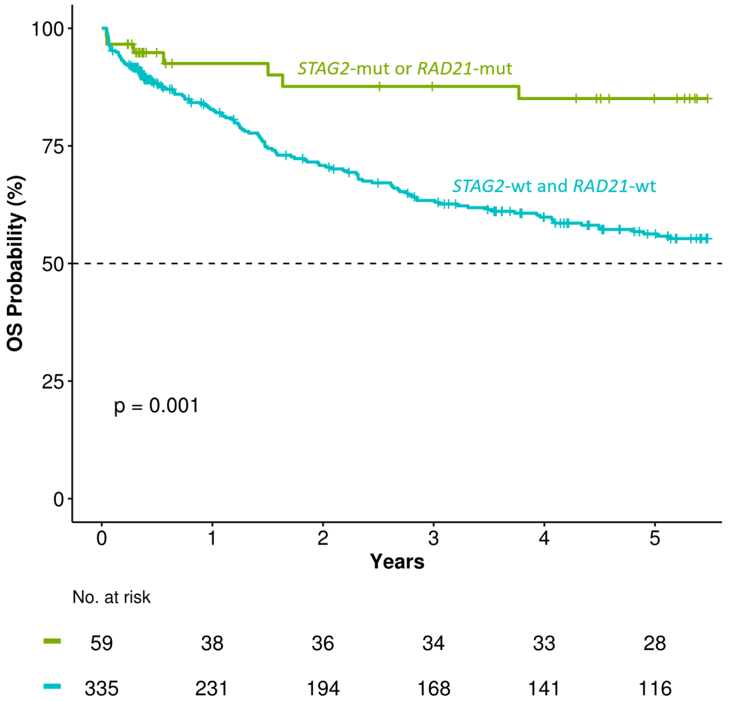

Figure S25. *STAG2* and *RAD21* mutations impact on overall survival in the subset of the training cohort with absence of *FLT3*-ITD.

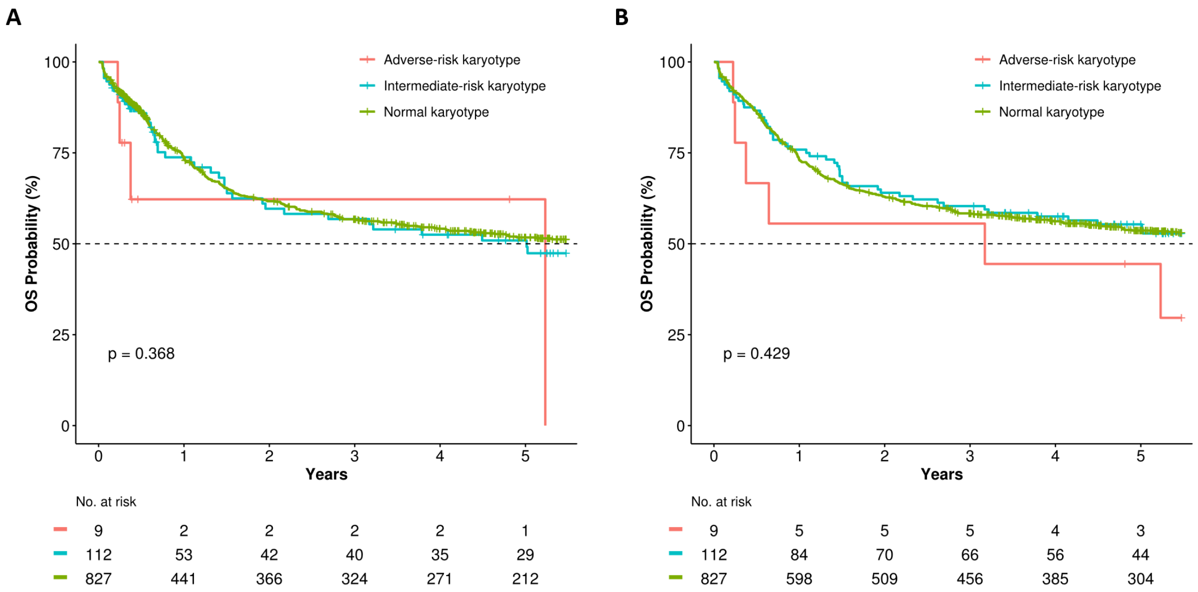

Figure S26. Overall survival of patients with *NPM1*-mut AML in the training cohort, stratified by cytogenetic aberrations, censoring patients that underwent allo-HSCT in CR1 at the transplant date (**A**) and without censoring transplanted patients (**B**).

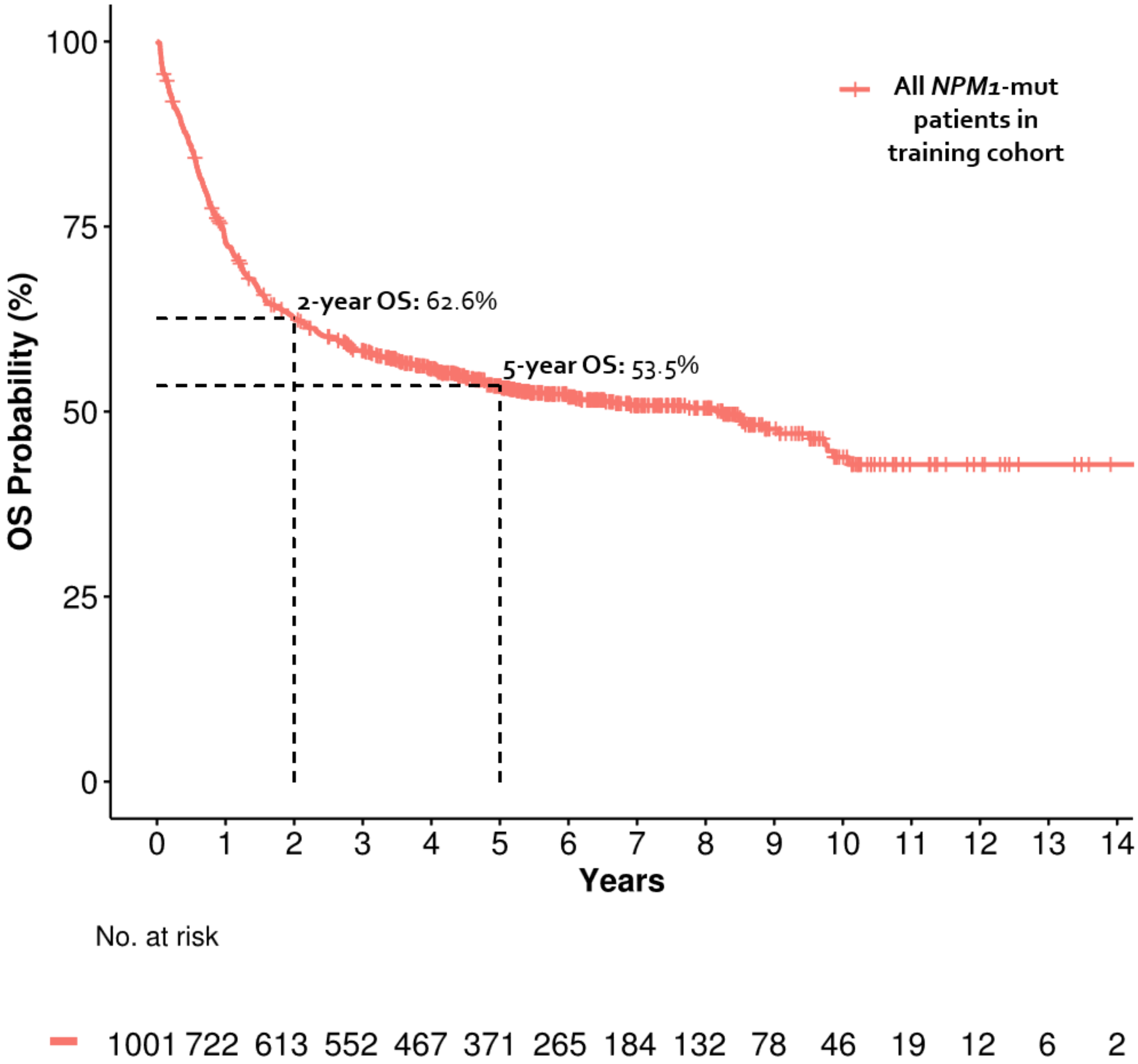

Figure S27. Overall survival of *NPM1*-mut patients in training cohort.
